# Computed Tomography Imaging Guided Microenvironment‐Responsive Ir@WO_3−x_ Dual‐Catalytic Nanoreactor for Selective Radiosensitization

**DOI:** 10.1002/advs.202405192

**Published:** 2024-08-05

**Authors:** Jiayu Song, Yue Feng, Jiazhuo Yan, Ying Wang, Weixiao Yan, Nan Yang, Tusheng Wu, Sijia Liu, Yuan Wang, Nannan Zheng, Liangcan He, Yunyan Zhang

**Affiliations:** ^1^ Department of Gynecological Radiotherapy Harbin Medical University Cancer Hospital Harbin 150001 China; ^2^ School of Medicine and Health Key Laboratory of Microsystems and Microstructures Manufacturing Harbin Institute of Technology Harbin 150001 China; ^3^ Department of Gynecological Oncology Zhejiang Cancer Hospital Zhengzhou Zhejiang 310022 China; ^4^ Zhengzhou Research Institute Harbin Institute of Technology Zhengzhou Henan 450000 China

**Keywords:** dual catalytic activity, hypoxia relief, nanoreactors, radiotherapy, synergistic therapies

## Abstract

Radiotherapy (RT) is often administered, either alone or in combination with other therapies, for most malignancies. However, the degree of tumor oxygenation, damage to adjacent healthy tissues, and inaccurate guidance remain issues that result in discontinuation or failure of RT. Here, a multifunctional therapeutic platform based on Ir@WO_3−x_ is developed which simultaneously addresses these critical issues above for precision radiosensitization. Ir@WO_3−x_ nanoreactors exhibit strong absorption of X‐ray, acting as radiosensitizers. Moreover, ultrasmall Ir enzyme‐mimic nanocrystals (NCs) are decorated onto the surface of the nanoreactor, where NCs have catalyst‐like activity and are sensitive to H_2_O_2_ in the tumor microenvironment (TME) under near infrared‐II (NIR‐II) light stimulation. They efficiently catalyze the conversion of H_2_O_2_ to O_2_, thereby ameliorating hypoxia, inhibiting the expression of HIF‐1α, and enhancing RT‐induced DNA damage in cancerous tissue, further improving the efficiency of RT. Additionally, in response to high H_2_O_2_ levels in TME, the Ir@WO_3−x_ nanoreactor also exerts peroxidase‐like activity, boosting exogenous ROS, which increases oxidative damage and enhances ROS‐dependent death signaling. Furthermore, Ir@WO_3−x_ can serve as a high‐quality computed tomography contrast agent due to its high X‐ray attenuation coefficient and generation of pronounced tumor‐tissue contrast. This report highlights the potential of advanced health materials to enhance precision therapeutic modalities.

## Graphical abstract

A TME‐responsive Ir@WO_3‐x_ multifunctional nanotherapeutic platform integrating tumor CT imaging and high‐efficiency radiotherapy is constructed. It can respond to the high expression of H_2_O_2_ in the TME, convert H_2_O_2_ to O_2_ through the catalytic activity of its own CAT‐like and POD‐like activity, ameliorating hypoxia, and promote ROS production, enhancing RT‐induced DNA damage, further improving the efficiency of radiotherapy.

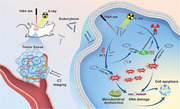

## 1 Introduction

As one of the most primary and irreplaceable therapeutic modalities in clinical practice, radiotherapy (RT) is applied to up to 60% of all malignant tumors at different stages, either as curative or palliative treatment.^[^ ^1^ ^]^ Given its extreme‐strength ionizing radiation, RT delivered via γ‐beam or x‐beam can cause oxidative stress and/or permanent DNA damage through the production of toxic cellular reactive oxygen species (ROS), ultimately leading to tumor ablation.^[^ ^2^ ^]^ However, the characteristic hypoxia of the tumor microenvironment (TME), driven by rapid tumor proliferation and increased O_2_ consumption due to vascular disorders, hampers the activation of radiation‐induced DNA repair pathways. This, in turn, promotes radiation resistance and undermines tumor eradication.^[^ ^3^ ^]^ Excessive radiation to address hypoxia, however, exacerbates the damage to adjacent healthy tissues. The clinical advent of imaging‐guided RT systems has facilitated the precise targeting of radiation doses in target areas, effectively reducing damage to normal tissues and organs while greatly contributing to the accurate treatment of malignancies.^[^ ^4^ ^]^ Although RT equipment has been optimized, the achievement of simultaneous radiosensitization and imaging quality for optimal RT efficacy remains a challenge. There is an urgent need for advanced radiosensitizers with sensitive imaging guidance to reverse hypoxia in TME and overcome radiation resistance, thereby improving outcomes for patients with advanced tumors.

With the significant development in nanobiotechnology platforms, high‐Z elements capable of reacting with tumors and attracting radiological beams (e.g., X‐ray) can serve as radiosensitizers. This enhances irradiation‐induced tumor damage by increasing energy deposition in tumors, while also providing guidance via computed tomography (CT)‐based imaging.^[^ ^5^ ^]^ We previously demonstrated the superiority of W_18_O_49_ and dual‐mode CT‐ or photoacoustic tomography (PAT)‐guided nano‐theranostics for radiosensitization.^[^ ^6^ ^]^ In addition, inspired by the complex tumor microenvironment characterized by hypoxia and high levels of H_2_O_2_, nanoreactors with selective radiotherapy sensitization have been extensively studied. The nanoreactors mimic biochemical reaction functions akin to peroxidase and catalase processes, and their activity is influenced by the tumor microenvironment to exert chemical and biological effects for radiosensitization.^[^ ^7^ ^]^ These enzymatically active nanoreactors stimulate enzymatic processes, exhibiting kinetics and mechanisms comparable to natural enzymes in physiological settings.^[^ ^8^ ^]^ TME, relative to healthy tissue environments, is generally characterized by overproduction of H_2_O_2_ and severe hypoxia, which play pivotal roles in tumor growth, metastasis, and treatment resistance.^[^ ^9^ ^]^ Enzymatically active nanoreactors exploit the TME properties for radiosensitization. For example, the abundance of oxygen vacancies on the surface of WO_3−x_ gives it catalytic activity comparable to peroxidase (POD), which can produce the toxic hydroxyl radicals (·OH) from H_2_O_2_ to eradicate cancer cells.^[^ ^6^, ^10^ ^]^ Notably, nanoreactors with catalase (CAT)‐like activity, such as ultrasmall Ir enzyme‐mimic nanocrystals (NCs),^[^ ^11^ ^]^ can dramatically alter tumor hypoxia, thereby sensitizing solid tumors to RT.^[^ ^12^ ^]^ A considerable number of enzymatically active nanoreactors have been discovered and reported, exhibiting various enzymatic activities.^[^ ^13^ ^]^ The combination of POD‐like and CAT‐like activities within a single nanoreactor serves as a powerful catalyst, facilitating the generation of O_2_ and ·OH for radiosensitization.^[^ ^14^ ^]^

**Scheme 1:**
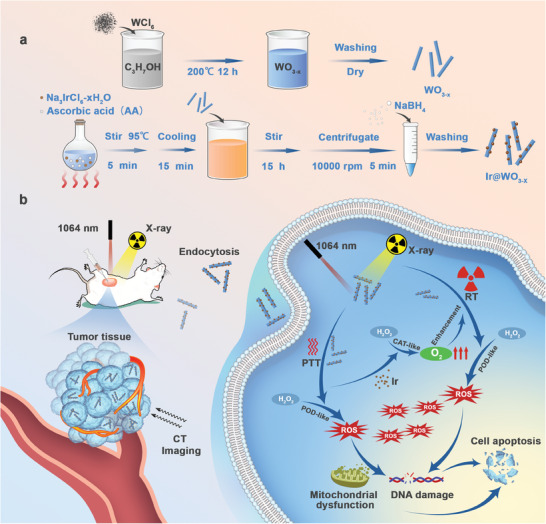
Schematic diagram of the Ir@WO_3−x_ nanoreactors a) and the mechanism by which it can be used for combined radiation and photothermal treatments b). Due to its peroxidase‐like activity, Ir@WO_3−x_ can induce the formation of extremely toxic ROS (e.g., ·OH) from H_2_O_2_ in response to the elevated levels of endogenous H_2_O_2_ in the tumor microenvironment during such treatments. Meanwhile, due to its catalase‐like activity, Ir@WO_3−x_ can break down H_2_O_2_ and release O_2_, which can reverse tumor microenvironment hypoxia and increase radiation sensitivity.

In this study, we designed a therapeutic Ir@WO_3−x_ nanoreactor with O_2_ sensitivity for selective radiotherapy (**Scheme** **1**). This nanoplatform comprises two high‐Z elements (W and Ir), enabling tumor‐specific radio enhancement with a high X‐ray attenuation capacity. The in situ reduction of ultrasmall Ir NCs on the WO_3−x_ surfaces enables significant reversal of tumor hypoxia‐induced radioresistance. The WO_3−x_ surface contains abundant O_2_ vacancies, which excite free electrons in the conduction band, and its substantial absorbance over the whole near‐infrared (NIR: 780–1100 nm) region, making it conductive to photothermal conversion. When coupled with a NIR laser, Ir@WO_3−x_ triggers a relatively controllable irritation response system, releasing O_2_ on demand for use in O_2_‐based therapies that enhance tumor‐specific radiation. Moreover, X‐ray provides a major energy source for clinical RT, overcoming the spatial and temporal limitations in tissue penetration depth inherent to conventional light‐activated therapies. They also effectively trigger the release of O_2_ and the generation of ROS, thereby enhancing the dual‐catalytic activity of nanoreactors and leading to more favorable therapeutic outcomes. Ir@WO_3−x_ is not only an excellent sensitizing agent for nano‐radiotherapeutic but also functions as a versatile agent for precise CT‐guided treatment. In this study, the Ir@WO_3−x_ has demonstrated efficient tumor restraint in vivo, suggesting that its coordinated catalysis by this nanoreactor holds promise for tumor treatment.

## 2 Results and Discussion

### 2.1 Synthesis and Characterization of Ir@WO_3−x_ Nanoreactors

To obtain Ir@WO_3−x_ nanoreactors with uniform rhomboid morphology, WO_3−x_ nanorods (NRs) were first synthesized (Figure S1, Supporting Information). To enable nanoparticles to circulate in the blood for an extended duration while avoiding rapid clearance by the kidneys, we employed an ultrasonic processor to decrease the size of WO_3−x_ NRs. Furthermore, the oxygen vacancies in the WO_3−x_ crystal lattice provided an environment for Ir^3+^, and electrostatic interaction allowed Ir^3+^ in solution to be adsorbed on the negatively charged WO_3−x_ NR surfaces. After Ir NCs were in situ prepared on the WO_3−x_ surfaces using NaBH_4_ as a reducing agent, yielding Ir@WO_3−x_ nanoreactors for multifunctional radiosensitization.

**Figure 1:**
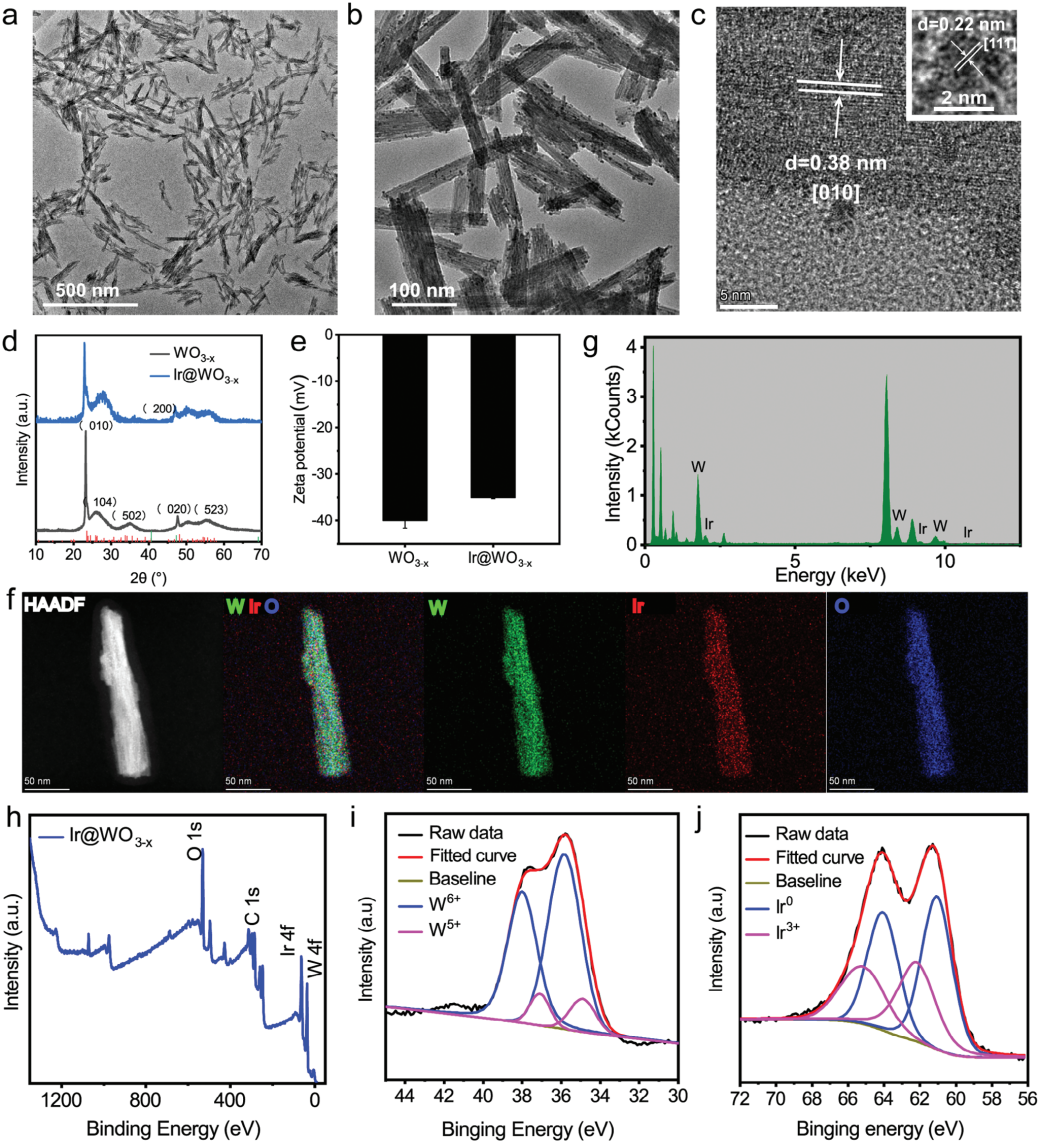
Nanoreactor characterization. a) TEM image of WO_3−x_ after ultrasonic fracture. b) TEM image of Ir@WO_3−x_. c) High‐resolution TEM image of Ir@WO_3−x_. d) XRD pattern of Ir@WO_3−x_. e) Zeta potential of Ir@WO_3−x_ (mean ± SD, *n =* 3). f) HAADF‐STEM image and element mapping of Ir@WO_3−x_. g) EDS spectra of Ir@WO_3−x_. h) XPS spectra of Ir@WO_3−x_ nanoreactors. XPS spectra of W 4f i) and Ir 4f j) core levels in Ir@WO_3−x_ nanoreactors.

As we expected, TEM confirmed that after ultrasonic processing, the lengths and widths of WO_3−x_ NRs became shorter from 346.4 ± 9.5 and 53.1 ± 3.0 nm to 133.2 ± 1.0 and 15.0± 0.3 nm, respectively (**Figure** **1**a; Figure S2, Supporting Information). The as‐prepared Ir@WO_3−x_ nanoreactors had average lengths and widths of 158.2 ± 3.9 and 20.6 ± 0.1 nm (Figure 1b; Figure S3, Supporting Information). The Ir@WO_3−x_ nanoreactors maintained a rod‐like shape, even after the in situ decoration of Ir NCs. Several ultrasmall individual Ir NCs domains were observed on their surfaces. High‐resolution TEM pictures revealed a well‐formed single crystal with a planar spacing of 0.38 nm, in agreement with the (010) crystal plane of monoclinic WO_3−x_. The crystal planar spacing of Ir was observed to be 0.22 nm, consistent with its (111) crystal plane (Figure 1c).^[^ ^15^ ^]^ In Figure 1d, it showed the XRD patterns of the Ir@WO_3−x_ nanoreactors. The as‐prepared WO_3−x_ NRs were monoclinic scheelite structures (JCPDS no. 712450). The diffraction peaks associated with the WO_3−x_ NRs [equivalent to (010)] gradually lost intensity with the addition of Ir.^[^ ^16^ ^]^ HAADF‐STEM imaging confirmed the presence of Ir, O, and W in the nanoreactors; an elemental map is shown in Figure 1f. HAADF‐STEM and EDS images showed that ultra‐small Ir NCs were successfully and evenly distributed on the WO_3−x_ NRs surfaces (Figure 1g). The nanoreactors possessed the desired atomic ratio of Ir, W, and O (Table S1, Supporting Information), which deviated from the stoichiometric ratio because of their anisotropic shapes.^[^ ^17^ ^]^

The chemical compositions of the key elements in the Ir@WO_3−x_ nanoreactors were analyzed further by XPS to gain a deeper understanding of the valence states of the compounds in the composites (Figure 1h–j). Ir, O, and W made up the majority of the X‐ray photoelectron spectrum of the Ir@WO_3‐x_ NRs, in line with previous element mapping.^[^ ^15^ ^]^ In agreement with previous findings, thorough analysis of the XPS peaks matching W revealed that the element was in a heterogeneous bonding state; W atoms with 6+ valences (W 4f 5/2 at 38.0 eV and W 4f 7/2 at 35.8 eV) were responsible for the primary peak. The W 4f 5/2 and W 4f 7/2 core levels from W^5+^ were responsible for the second doublet, which had lesser binding energies at 37.1 and 34.9 eV. These two main oxidation states had been noted in WO_3−x_ NRs. The Ir peaks were composed mainly of Ir^3+^ and fully reduced Ir elements, giving the Ir@WO_3−x_ nanoreactors good hydrogen peroxide activity. The O 1s spectra had three distinct peaks at 530.7, 531.7, and 532.5 eV, corresponding to lattice O_2_, O atoms closed to O_2_‐generated vacancies, and O_2_ adsorbed by hydroxyl groups on the nanomaterial surfaces, respectively (Figure S4, Supporting Information).^[^ ^16^ ^]^ The Ir@WO_3−x_ nanocomposite had a median hydrodynamic size of 206.8 ± 4.9 nm and a negative charge, which facilitated the endocytosis of cancer cells (Figure S5, Supporting Information).^[^ ^18^ ^]^ The in situ synthesis of Ir NCs also changed the charge of the zeta potential from −40.1 ± 1.6 mV for the WO_3−x_ NRs to −35.1 ± 0.2 mV (Figure 1e). In addition, stability tests showed that the Ir@WO_3−x_ nanoreactor had good dispersion and stability (Figure S6, Supporting Information). The size of the Ir@WO_3−x_ showed little variation in 72 h, facilitating its catalytic effect inside the tumor.

### 2.2 Photothermal Property of Ir@WO_3−x_ Nanoreactors

**Figure 2:**
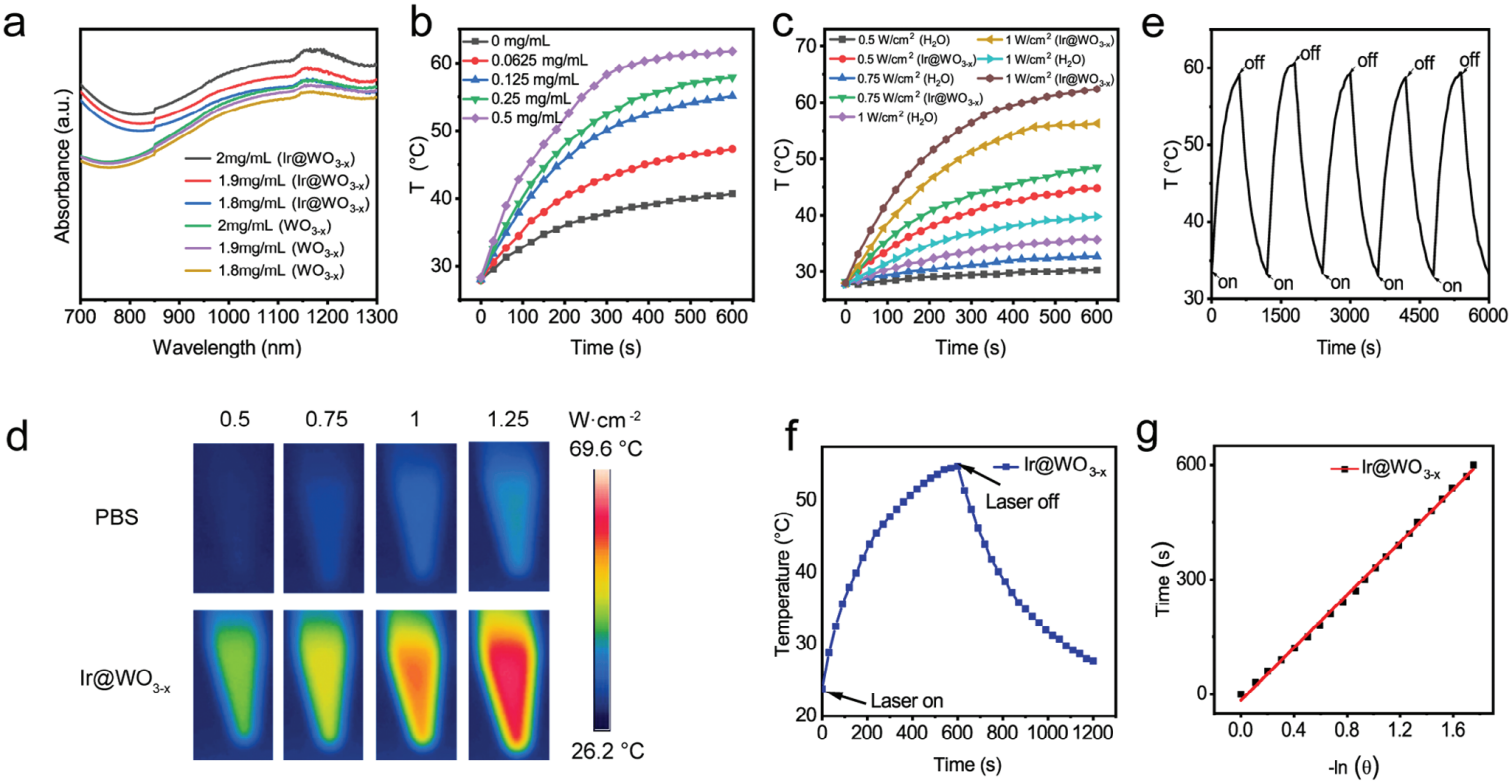
Photophysical characteristics and photothermal transformation effect of Ir@WO_3−x_ nanoreactors. a) UV–Vis–NIR absorption spectra for WO_3−x_ NRs and Ir@WO_3−x_ nanoreactors. b) Temperature increase curves for Ir@WO_3−x_ nanoreactors under 1064‐nm laser irradiation (1 W·cm^−2^). Temperature changes c) and thermograms of photothermal conversion d) for PBS and Ir@WO_3−x_ nanoreactors under 1064‐nm laser irradiation (1 W·cm^−2^). e) Photothermal stability of Ir@WO_3−x_ nanoreactors during five on/off cycles of 1064‐nm laser irradiation (1 W·cm^−2^). f) Heating and cooling curves for Ir@WO_3−x_ nanoreactors under 1064‐nm laser irradiation (1 W·cm^−2^). g) Efficiency of photothermal conversion, determined from the cooling cycle.

The visible‐NIR absorbance spectrum of the Ir@WO_3−x_ nanoreactors fully encompassed the targeted biological‐NIR “first and second windows” (700‐950 and 1000–1350 nm, respectively), with wide and robust absorption in the NIR region (**Figure** **2**a).^[^ ^19^ ^]^ The NIR absorption characteristic of the Ir@WO_3−x_ nanoreactors was caused by localized surface plasmon resonance (LSPR) absorption due to the O_2_ vacancies in the crystal lattice and an abundance of liberated electrons.^[^ ^20^ ^]^ Additionally, the absorbance of the Ir@WO_3‐x_ nanoreactors was increased markedly in the concentration‐dependent experiments than that of WO_3‐x_, as the addition of Ir NCs at the same concentration of the WO_3‐x_ NRs.

Given the efficient optical absorption observed in the NIR region, the photothermal conversion capability of Ir@WO_3−x_ nanoreactors in PBS under NIR laser irradiation was evaluated. The influence of concentration on temperature rise showed that the temperature increase achieved by laser irradiation at 1064 nm with NIR‐II was better than that achieved by laser irradiation at 808 nm with NIR‐I, and the Ir@WO_3−x_ nanoreactors quickly and effectively transformed laser energy into localized hyperthermia. Therefore, for subsequent experiments, we chose to irradiate with a 1064 nm laser in the NIR‐II region. The photothermal conversion capability of Ir@WO_3−x_ nanoreactors mainly depended strongly on the irradiation duration, nanoreactors concentration, and laser power density (Figure 2b–d; Figure S7, Supporting Information). A notable temperature increase was observed after 10 min exposure to 1 W·cm^−2^ radiation and the temperature of the 0.25 mg mL^−1^ Ir@WO_3−x_ nanoreactor solution increased much more (24.4 °C) than did the PBS solution (Figure 2b). In addition, under NIR‐II laser irradiation with a wavelength of 1064 nm and the same power, the photothermal heating effect of solution Ir@WO_3−x_ with the same concentration was better than that of solution WO_3−x_. At the 10th minute, the temperature increased by 5.4 °C, indicating that Ir@WO_3−x_ has a better photothermal effect than WO_3−x_ (Figure S8, Supporting Information). The temperature curves were very similar across five cycles, indicating that the nanomaterials have good photothermal stability (Figure 2e). The Ir@WO_3−x_ nanoreactors had a photothermal conversion efficiency (*η*) of 37.05% (Figure 2f,g).^[^ ^21^ ^]^ This high efficiency in the NIR range may enhance tumor reoxygenation via blood vessel dilation and increased intratumoral blood flow.^[^ ^14^ ^]^ Ir@WO_3−x_ nanoreactors thus have the potential to serve as anticancer PTT agents due to their exceptional photothermal conversion capability and excellent photothermal stability.

### 2.3 Dual‐enzyme Nanocascades of Ir@WO_3−x_ Nanoreactors for O_2_ and ·OH Generation In Vitro

The H_2_O_2_ concentration is substantially greater in the TME (100 µm‐1.0 mm) than in normal cells.^[^ ^22^ ^]^ X‐ray radiation has an ionizing effect, increasing H_2_O_2_ production and further contributing to high H_2_O_2_ levels in cancer cells.^[^ ^22^, ^23^ ^]^ Based on the literature and our previous work,^[^ ^6^, ^11^ ^]^ we hypothesized that Ir@WO_3−x_ nanoreactors would have high CAT‐like and POD‐like catalytic activity, transforming endogenous and exogenous H_2_O_2_ into O_2_ and ·OH, respectively. The Ir@WO_3−x_ nanoreactors possessed dual‐enzyme activity, enhancing the biological effects of RT by facilitating DNA damage through the accumulation of ·OH radicals. Additionally, they provided a controlled and efficient supply of O_2_, effectively mitigating tumor hypoxia‐induced radioresistance.

**Figure 3:**
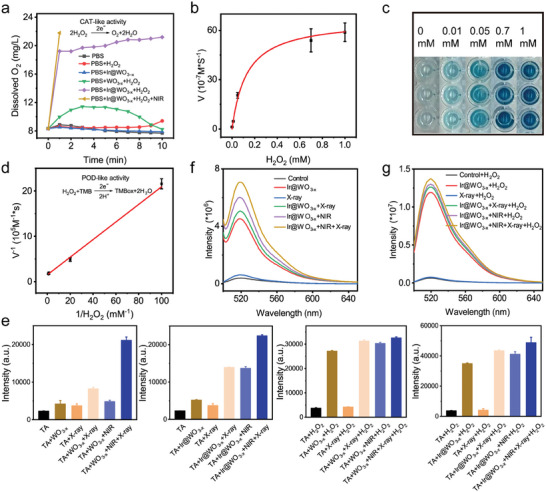
Catalase‐ and peroxidase‐like activities of Ir@WO_3−x_ nanoreactors. a) Oxygen production profiles for WO_3−x_ NRs and Ir@WO_3−x_ nanoreactors in H_2_O_2_ solution (0.1 mm), measured using a portable dissolved oxygen meter. b) oxTMB absorbance of Ir@WO_3−x_ nanoreactors peroxidase mimetics with TMB substrate and various H_2_O_2_ concentrations (mean ± SD, *n =* 3). c) Color variation of samples from (b). d) Linear fit of H_2_O_2_ concentrations to oxTMB absorbance at 652 nm (mean ± SD, *n =* 3). e) Fluorescence spectra of ·OH groups, detected by TA (mean ± SD, *n =* 3). ROS fluorescence spectra in the Ir@WO_3−x_ nanoreactors f) and Ir@WO_3−x_ nanoreactors + H_2_O_2_ g) groups, detected by DCFH.

A dissolved O_2_ meter was used to monitor O_2_ production and thereby determine whether the Ir@WO_3−x_ nanoreactors had CAT‐like activity. After 10 min in the simulated TME with optimal pH (6.5), the Ir@WO_3−x_ nanoreactors produced more than 2.6 times more dissolved O_2_ than the WO_3−x_ NRs with the same molar mass (**Figure** **3**a). The presence of Ir clearly enhanced the ability of the nanomaterials to decompose H_2_O_2_, accelerating O_2_ generation and resulting in superior catalytic performance of the Ir@WO_3−x_ nanoreactors. When exposed to NIR‐II radiation, the Ir@WO_3−x_ nanoreactors were also at a higher temperature due to the photothermal effect, which dramatically accelerated the rate of O_2_ synthesis from H_2_O_2_ solution [21.79 mg L^−1^ in 1 min and >22.0 mg L^−1^ (the upper limit of meter detection) in 2 min; Figure 3a]. Given this NIR‐induced photocatalytic‐mediated H_2_O_2_ decomposition, the Ir@WO_3−x_ nanoreactors efficiently and relatively controllably provided an O_2_ supply for the clinical alleviation of tumor hypoxia and enhancement of radiosensitization.

The POD‐like activity of the Ir@WO_3−x_ nanoreactors was assessed in vitro. The ability of the Ir@WO_3−x_ nanoreactors to generate ·OH was estimated using TMB as a substrate at 37 °C (pH = 6.5) when the range of detected concentration of H_2_O_2_ was reduced to be equal to or lower than TME.^[^ ^14^ ^]^ The absorption coefficient increased with the H_2_O_2_ concentration (0, 0.01, 0.05, 0.1, 0.7, and 1 mm; Figure 3b), consistent with the color variation shown in Figure 3c. The *R* ^2^ value (0.9974) reflected a good fit along the linear spectrum from 0 to 1 mm, indicating that the Ir@WO_3−x_ nanoreactors had POD‐like activity (Figure 3d). Therefore, the Ir@WO_3−x_ nanoreactors exhibited strong POD‐like catalytic activity, catalyzing H_2_O_2_ breakdown in the H_2_O_2_‐enriched TME. Besides, regarding the Km and Vmax values, the Km value of the Ir@WO_3−x_ nanoreactors for TMB substrates was several times lower than that of horseradish peroxidase (0.130 versus 0.434 mm), providing direct evidence of the high substrate affinity of the Ir@WO_3−x_ nanoreactors (Table S2, Supporting Information).

Terephthalic acid (TA) results confirmed the occurrence of an ·OH burst when the Ir@WO_3−x_ nanoreactors were exposed to both internal (H_2_O_2_) and external (X‐ray and/or NIR‐II irradiation) stimulation.^[^ ^24^ ^]^ The Ir@WO_3−x_ nanoreactors rapidly increased the 2‐hydroxyterephthalic acid (TAOH) fluorescence signal to surpass the signal obtained with the WO_3−x_ NRs (Figure 3e). This result indicated that the Ir@WO_3−x_ nanoreactors possess POD‐like activity. We confirmed the X‐ray treatment of Ir@WO_3−x_ nanoreactors induced an ·OH burst, likely attributable to the capture of low‐energy electrons and holes induced by X‐ray in the conduction and valence bands, respectively, of the semiconductor nanomaterials.^[^ ^25^ ^]^ As WO_3−x_ efficiently converted NIR‐II energy into heat, facilitating the catalytic response, via the LSPR effect,^[^ ^26^ ^]^ the results in Figure 3e showed that the Ir@WO_3−x_ nanoreactors selectively converted excess intratumoral H_2_O_2_ into highly oxidized ·OH and that NIR‐II irradiation enhanced their POD‐like catalytic efficiency. Under X‐ray irradiation, semiconductor nanomaterials’ valence and conduction bands had been shown to capture low‐energy electrons and holes, respectively, encouraging ·OH production. Together, these results demonstrated that the Ir@WO_3−x_ nanoreactors exhibit high POD‐like activity that enhances the effects of RT.

The Ir@WO_3−x_ nanoreactors also accelerated efficient ROS generation by responding to H_2_O_2_. The absorbance rose significantly in the presence of Ir@WO_3−x_ nanoreactors but only slightly in the presence of WO_3−x_ NRs (Figure 3f; Figure S9a, Supporting Information). Under 1064‐nm NIR‐II irradiation, the ROS yield from the Ir@WO_3−x_ nanoreactors was 1.33 times higher than that from the WO_3−x_ NRs, particularly when the nanoreactors were co‐irradiated with NIR and X‐ray, where the highest ROS generation was observed, reflecting strong POD‐like activity. The Ir@WO_3−x_ nanoreactors catalyzed the decomposition of excess H_2_O_2_ (100 µm–1.0 mm) into highly toxic ROS in hypoxic tumors. In the presence of H_2_O_2_, the fluorescence of ROS was further enhanced by X‐ray/NIR‐II irradiation (Figure 3g; Figure S9b, Supporting Information). The ROS production of the Ir@WO_3−x_ nanoreactors was greatest with combined NIR‐II and X‐ray irradiation. Taken together, these findings demonstrated that the Ir@WO_3−x_ nanoreactors enable the utilization of overexpression in hypoxic tumors to generate O_2_‐independent free radicals, thereby improving efficacy.

### 2.4 In Vitro Therapeutic Efficacy

**Figure 4:**
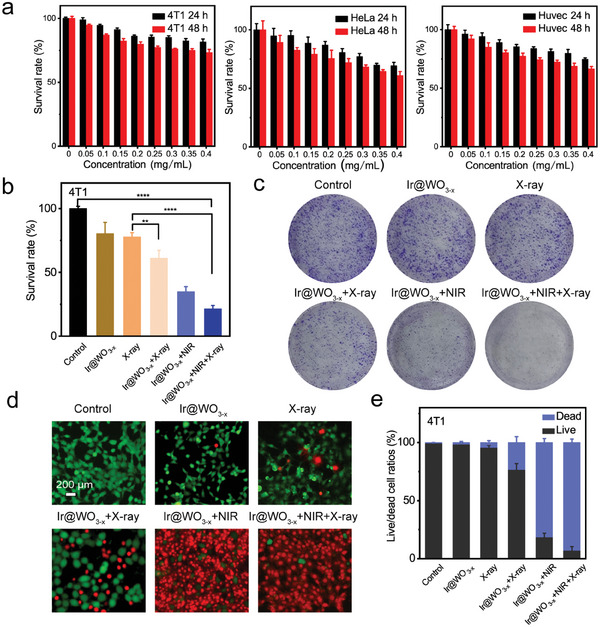
In vitro radiotherapy sensitization of Ir@WO_3−x_ nanoreactors. a) Cytotoxicity of Ir@WO_3−x_ nanoreactors to 4T1 cells, HeLa cells, and HUVECs after 24 and 48 h (mean ± SD, *n =* 6). b) 4T1 cell survival with various treatments (mean ± SD, *n =* 4). c) Photographs of 4T1 cell colony formation in various treatment groups. Calcein‐AM/PI fluorograms d) and statistics for 4T1 cells in various treatment groups e) (mean ± SD, *n =* 3). *P* values were assessed using a two‐tailed Student's t‐test with GraphPad Prism 8.0. **p < 0.01, ****p < 0.0001.

The cellular uptake and biosafety of the Ir@WO_3−x_ nanoreactors as radiosensitizers were examined in 4T1 and HeLa cells (**Figure** **4**). Bio‐TEM images showed that the Ir@WO_3−x_ nanoreactors were incorporated in the cytoplasm of 4T1 cells after 12 h co‐incubation (Figure S10, Supporting Information).^[^ ^27^ ^]^ Inductively coupled plasma‐optical emission spectrometry (ICP‐OES) analysis showed that the Ir and W contents per 10^5^ cells increased to ≈2.35 and 34.52 µg, respectively, after 12 h incubation with 0.25 mg mL^−1^ nanoreactors and 24 h culture (Figure S11, Supporting Information). CCK‐8 assays revealed no significant concentration‐dependent cytotoxicity of the Ir@WO_3−x_ nanoreactors for 4T1 cells, HeLa cells, or human umbilical‐vein endothelial cells (HUVECs), with cell viability exceeding 80% in all groups (Figure 4a). CCK‐8 assays were also performed after RT, with 6 Gy selected as the optimal dose (Figure S12, Supporting Information). The maximum 4T1 and HeLa cell killing effects were observed with X‐ray and NIR‐II irradiation (Figure 4b; Figure S13, Supporting Information). Notably, lower radiation doses could be used to achieve good RT effects without damaging normal tissues. These results indicated that the X‐ray and NIR‐II irradiation of Ir@WO_3−x_ nanoreactors significantly and controllably increases ·OH production, which could improve the efficacy of targeted RT in clinical settings.

The radiotherapeutic efficacy of the Ir@WO_3−x_ nanoreactors was further evaluated in tumor cells. Initially, the radiosensitizing effect of the Ir@WO_3−x_ nanoreactors was observed visually using a clonogenic assay. Separate exposure to X‐ray and NIR‐II laser irradiation with Ir@WO_3−x_ nanoreactors reduced the numbers of viable 4T1 cell colonies to 111 and 310, respectively, relative to a count of 501 with exposure to X‐ray radiation without Ir@WO_3−x_ nanoreactors. With Ir@WO_3−x_ nanoreactors and combined X‐ray and NIR‐II laser irradiation, the viable cell colony count was effectively reduced to 54 (Figure 4c). These results indicated that the Ir@WO_3−x_ nanoreactors were highly promising photothermal agents and radiosensitizers, with a combined beneficial impact that exceeded their separate effects. Live/dead cell staining yielded similar results, confirming that the Ir@WO_3−x_ nanoreactors increased 4T1 cell apoptosis with X‐ray irradiation (Figure 4d,e). The achievement of the lowest 4T1 cell survival rate in the Ir@WO_3−x_ nanoreactors + NIR‐II + X‐ray irradiation group could be attributed to O_2_ and ROS production in the TME. To further ascertain the radiotherapeutic efficacy of the Ir@WO_3−x_ nanoreactors, we conducted the above‐described experiments with HeLa cells and observed good radiosensitization ability (Figures S14 and S15, Supporting Information). These findings from different tumor cell lines suggested that the Ir@WO_3−x_ nanoreactors were universally able to achieve improved tumor RT through X‐ray–induced ROS production.

### 2.5 Hypoxic Regulation In Vitro

**Figure 5:**
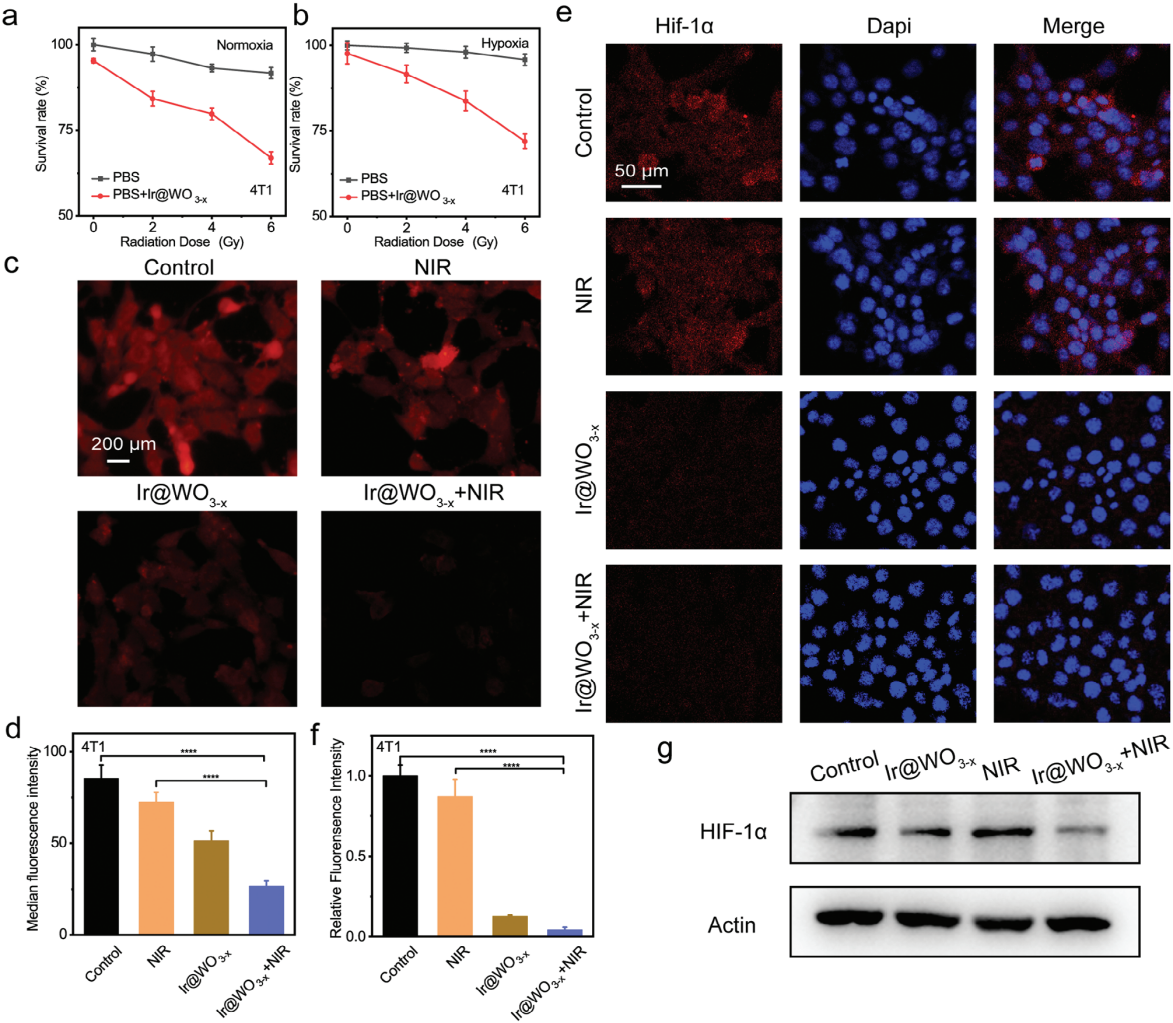
In‐vitro hypoxia regulation by Ir@WO_3−x_ nanoreactors. Cytotoxicity in 4T1 cells treated with Ir@WO_3−x_ nanoreactors under normoxia a) and hypoxia b) conditions, followed by X‐ray irradiation (mean ± SD, *n =* 6). c) Intracellular fluorescence intensity and statistics for [Ru(dpp)_3_]Cl_2_ in 4T1 cells in different treatment groups and corresponding statistics d) (mean ± SD, *n =* 3). HIF‐1α expression in 4T1 cells from various treatment groups e) and corresponding statistics f) (mean ± SD, *n =* 3). g) Western blot detection of HIF‐1α expression in 4T1 cells in different treatment groups. P values were assessed using a two‐tailed Student's t‐test with GraphPad Prism 8.0. ****p < 0.0001.

Based on the O_2_ generation of the Ir@WO_3−x_ nanoreactors, their hypoxic tumor‐specific radiosensitizing effect was investigated at the cellular level. First, 4T1 cells were exposed to normoxic and hypoxic conditions to examine the Ir@WO_3−x_ nanoreactors' cytotoxic effects. Cell viabilities in the X‐ray‐only groups and Ir@WO_3−x_ nanoreactors treatment groups correlated positively with the radiation dose under normoxic conditions (**Figure** **5**a). However, under hypoxic conditions, due to the inability of hypoxic cells to generate significant free radicals of O_2_ and the difficulty in fixing DNA damage by O_2_. Therefore, the cell survival rate of the pure X‐ray group did not change significantly (Figure 5b).^[^ ^28^ ^]^ In the case of HeLa cells, similar results were obtained (Figure S16, Supporting Information). To identify the potential of the Ir@WO_3−x_ nanoreactors to modulate intracellular hypoxic conditions, O_2_ content was measured with [Ru(dpp)_3_]Cl_2_.^[^ ^29^ ^]^ Relative to the control, hypoxia was alleviated in 4T1 cells treated with Ir@WO_3−x_ nanoreactors for 12 h, and barely red fluorescence was observed in 4T1 cells in the Ir@WO_3−x_ nanoreactors + NIR‐II irradiation group (Figure 5c,d). Experiments performed with HeLa cells yielded similar results. Taken together, these results confirmed that the Ir@WO_3−x_ nanoreactors have outstanding CAT‐like activity, reversing hypoxia‐related radioresistance following the intracellular O_2_ supply by NIR‐II photocatalysis (Figure S17, Supporting Information).

HIF‐1α is a marker that reflects intracellular O_2_ levels.^[^ ^30^ ^]^ Reduced HIF‐1α fluorescence was observed in cells incubated with Ir@WO_3−x_ nanoreactors and 1064‐nm NIR‐II irradiation, consistent with the above results and reflecting the hypoxia‐relieving CAT‐like activity of the Ir@WO_3−x_ nanoreactors (Figure 5e,f). According to the previous reports, under hypoxic conditions, HIF‐1 α production may promote angiogenesis and consequently inhibit vascular endothelial growth factor (VEGF) expression.^[^ ^31^ ^]^ We performed western blotting to investigate post‐treatment HIF‐1α expression in 4T1 cells at different timepoints. The Ir@WO_3−x_ nanoreactors caused a marked and dose‐dependent reduction of the HIF‐1α level due to the enrichment of intracellular O_2_ levels via their CAT‐like activity (Figure S18, Supporting Information). Moreover, HIF‐1α expression was significantly lower in the Ir@WO_3−x_ nanoreactors + NIR‐II irradiation group than in the Ir@WO_3−x_ nanoreactors group (Figure 5g). As excess H_2_O_2_ is produced in TME but not in normal tissues,^[^ ^32^ ^]^ Ir@WO_3−x_ nanoreactors + X‐ray treatment might be a viable approach to the enhancement of targeted anticancer RT efficiency. Taken together, these findings indicated that the Ir@WO_3−x_ nanoreactors function as photothermal conversion agents and boosted their own CAT‐like catalytic function for radiosensitization.

### 2.6 Mechanism of Ir@WO_3−x_ Nanoreactors Biological Radiosensitization

**Figure 6:**
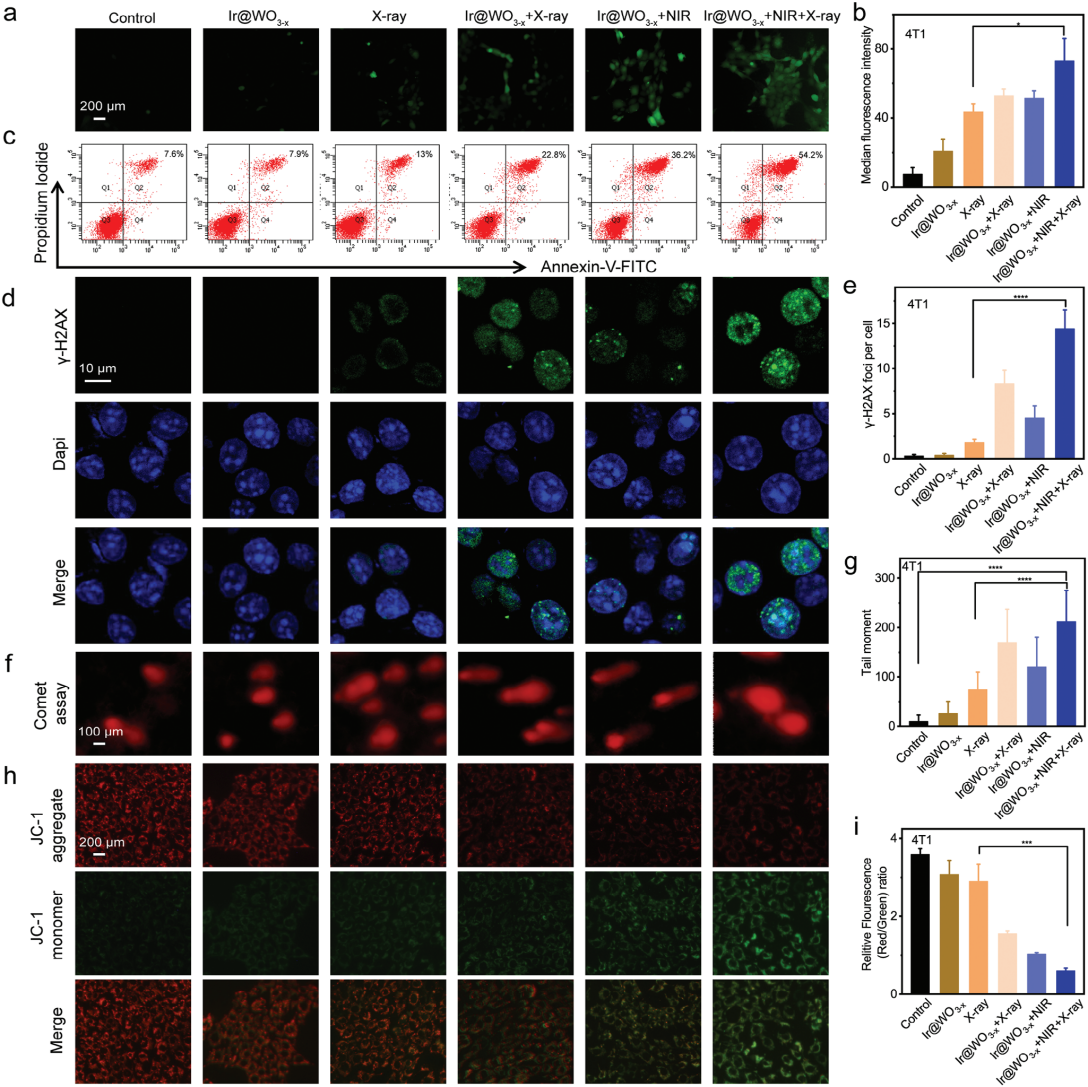
Analysis of Ir@WO_3−x_ nanoreactor radiosensitization mechanisms. Fluorescence images a) and statistical evaluation b) of ROS production by 4T1 cells under various conditions (mean ± SD, *n =* 3). c) Annexin V‐FITC/PI staining of 4T1 cell apoptosis under various conditions. Fluorescence images d) and statistical analysis e) of γ‐H2AX levels in 4T1 cells in different treatment groups (mean ± SD, *n =* 3). Comet assay f) and quantitative g) results for DNA damage in 4T1 cells under various conditions (mean ± SD, *n =* 3). JC‐1 assay h) and quantitative i) results for the mitochondrial membrane potential in 4T1 cells in different treatment groups (mean ± SD, *n =* 3). P values were assessed using a two‐tailed Student's t‐test with GraphPad Prism 8.0. *p < 0.05, ***p < 0.001, ****p < 0.0001.

Given our observation that the Ir@WO_3−x_ nanoreactors enhanced antitumor O_2_ generation and intracellular oxidation damage, we examined the mechanism of the nanoreactors’ biological radiosensitization in greater detail. ROS formation can induce direct or indirect DNA damage, which ultimately determines the therapeutic impact of radiation.^[^ ^33^ ^]^ The 2',7'‐dichlorofluorescein (DCF) fluorescence intensities in the Ir@WO_3−x_ nanoreactors + X‐ray irradiation, Ir@WO_3−x_ nanoreactors + NIR‐II irradiation, and Ir@WO_3−x_ nanoreactors + NIR‐II + X‐ray irradiation groups were, respectively, 45.37%, 43.7%, and 65.51% higher than that of the control. X‐ray–treated cells showed only very faint green fluorescence. These results indicated that the Ir@WO_3−x_ nanoreactors convert H_2_O_2_ into extremely hazardous ROS under NIR‐II or X‐ray irradiation (**Figure** **6**a,b). Using flow cytometry and annexin V–FITC/PI, cell apoptosis after the various treatments were examined. More apoptotic and necrotic 4T1 cells were observed after the Ir@WO_3−x_ nanoreactors + NIR‐II + X‐ray irradiation treatment than after the Ir@WO_3−x_ nanoreactors + X‐ray irradiation and X‐ray irradiation alone treatments (Figure 6c), suggesting that the former treatment induced spontaneous programmed cell death. In the same experiments conducted with HeLa cells, the greatest ROS production and highest apoptosis rate were also observed in the Ir@WO_3−x_ nanoreactors + NIR‐II + X‐ray irradiation group (Figures S19 and S20, Supporting Information). These results demonstrated that the Ir@WO_3−x_ nanoreactors were effective nanoradiosensitizers due to the simultaneous induction of ROS production and greater cell damage upon RT administration.

As ROS‐induced oxidative damage to DNA and mitochondria might explain the ability of the Ir@WO_3−x_ nanoreactors to increase radiotherapeutic efficiency,^[^ ^34^ ^]^ the nanoreactors’ tumor cell‐killing effects with combined treatment were assessed. Immunofluorescent labeling with γ‐H2AX was used to assess double‐strand DNA breakage.^[^ ^35^ ^]^ The highest γ‐H2AX level in 4T1 cells, reflecting increased DNA damage, was detected in the Ir@WO_3−x_ nanoreactors + NIR‐II + X‐ray irradiation group (Figure 6d,e). This result confirmed that the Ir@WO_3−x_ nanoreactors have CAT‐like activity, functioning as radiosensitizers due to the induction of DNA damage, supporting the O_2_ fixation hypothesis and validating the in‐vivo O_2_ abundance in hypoxic cancer cells.^[^ ^28a^ ^]^ The quantification of DNA damage by comet assay yielded similar results; more double‐strand DNA breaks were observed in the Ir@WO_3−x_ nanoreactors + NIR‐II + X‐ray irradiation group (*n =* 212.54) than in the Ir@WO_3−x_ nanoreactors + X‐ray irradiation (*n =* 169) and X‐ray irradiation alone (*n =* 74) groups (Figure 6f,g). Additionally, the breakdown of mitochondria due to decreased mitochondrial membrane potential is caused by the enhancement of oxidative damage in the cancer cells.^[^ ^36^ ^]^ JC‐1, a fluorescent probe for observing mitochondrial membrane potential, was used to assess the cell injury caused by ROS. The Ir@WO_3−x_ nanoreactors + X‐ray irradiation group showed the greatest reduction in mitochondrial membrane potential in 4T1 cells (Figure 6h,i), indicating that RT effectively promoted mitochondrial dysfunction and DNA damage by causing O_2_ and ROS production. Similar results were obtained with HeLa cells (Figures S21–S23, Supporting Information). Taken together, these results demonstrated that the Ir@WO_3−x_ nanoreactors efficiently increased DNA damage and double‐strand breakage and reduced the mitochondrial membrane potential in tumor tissues under X‐ray treatment, thereby achieving radiosensitization.

### 2.7 CT Features of Ir@WO_3−x_ Nanoreactors

**Figure 7:**
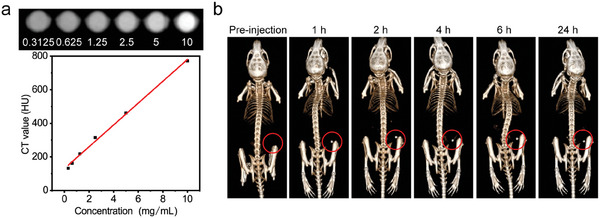
CT imaging of Ir@WO_3−x_ nanoreactors. a) Curve of Ir@WO_3−x_ nanoreactors solution concentrations versus CT values. From left to right: 0.3125; 0.625; 1.25; 2.5; 5; 10 mg mL^−1^. b) CT images were acquired at different timepoints after the intratumoral injection of Ir@WO_3−x_ nanoreactor solution (5 mg·kg^−1^). Significant enhancement of CT contrast is visible within the red dashed circle.

As the high‐Z elements of the Ir@WO_3−x_ nanoreactors (Ir, 77; W, 74) have high X‐ray attenuation coefficients, we investigated the potential of such nanoreactors as smart contrast agents for CT imaging. The CT signal strength increased with the Ir@WO_3−x_ nanoreactors concentration in vitro. An excellent linear relationship was observed between the Ir@WO_3−x_ nanoreactors' mass concentrations and the Hounsfield units (HU), with a slope value of 63.4 (**Figure** **7**a). The CT imaging potential of intratumoral injection of Ir@WO_3−x_ was assessed in vivo using 4T1‐BALB/c mice. CT showed only normal bone structures in the absence of Ir@WO_3−x_ nanoreactors, but robust contrast at tumor sites with intratumoral Ir@WO_3−x_ nanoreactors injection, demonstrating the radiosensitizers build‐up in tumors and the potential of Ir@WO_3−x_ nanoreactors for CT imaging (Figure 7b). The CT signal peaked 2 h after intratumoral injection and weakened at the tumor site after 24 h, reflecting a trend towards clearance of Ir@WO_3−x_ (Figure S24, Supporting Information). No CT signal was observed in the spleen or liver following the intratumoral nanoreactor injection. These CT results demonstrated that intratumorally injected Ir@WO_3−x_ nanoreactors evade high absorption by macrophages in reticuloendothelial system organs and accumulate in tumors, thereby reducing negative impacts on other organs.^[^ ^37^ ^]^ Thus, intratumoral administration may be the best option for the efficient achievement of contrast agents and sensitizer accumulation in tumors for safe, efficient radiosensitization.

### 2.8 In vivo Synergetic Oncotherapy

**Figure 8:**
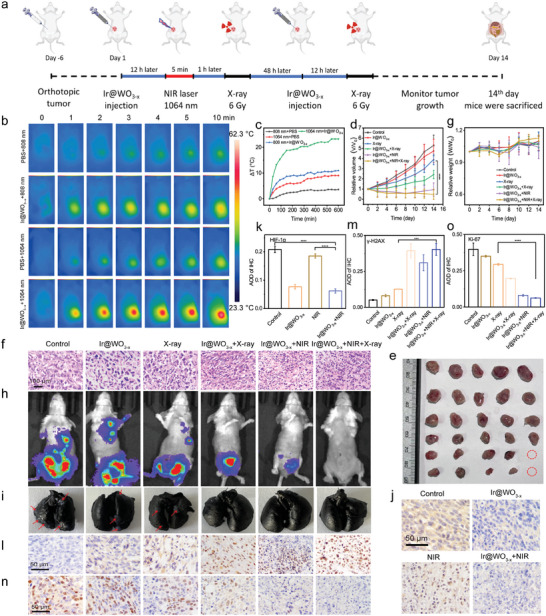
In vivo antitumor effects of Ir@WO_3−x_ nanoreactors. a) Schematic of combined Ir@WO_3−x_ nanoreactor use and RT/PTT. b) Infrared thermal images acquired after the treatment of mice with 4T1 breast cancer with 1064 nm laser irradiation and the intratumoral injection of PBS and Ir@WO_3−x_ nanoreactors. c) Curves of tumor‐site temperature increase after Ir@WO_3−x_ nanoreactor and PBS injection. Corresponding tumor growth curves d) and Representative photographs e) from different treatment groups (mean ± SD, *n =* 5). f) H&E‐stained tumor sections obtained 14 days after treatment. g) Body weight changes by treatment groups (mean ± SD, *n =* 5). h) Luciferase‐based imaging of tumor metastasis in the treatment groups after 28 days. i) Photographs of lung tumor nodules after 28 days of treatment. Immunohistochemical detection of HIF‐1α protein expression pin tissues j) and statistical analysis k) (mean ± SD, *n =* 3). Immunohistochemical detection of γ‐H2AX protein expression in tissues l) by IHC and statistical analysis m) (mean ± SD, *n =* 3). Immunohistochemical detection of Ki‐67 protein expression in tissues n) and statistical analysis o) (mean ± SD, *n =* 3). *P* values were assessed using a two‐tailed Student's t‐test with GraphPad Prism 8.0. ***p < 0.001, ****p < 0.0001.

We evaluated the biosafety of the Ir@WO_3−x_ nanoreactors for biomedical applications prior to radiosensitization treatment in vivo. Based on CT imaging and biological distribution studies, and to minimize adverse effects on organs, we administered Ir@WO_3−x_ nanoreactors into tumors in BALB/c mice. Routine blood and biochemical indicators showed no obvious abnormality, providing initial evidence for the biological safety of the Ir@WO_3−x_ nanoreactors (Figures S25 and S26, Supporting Information). 7 days after injection of the Ir@WO_3−x_ nanoreactor solution, the mice were euthanized. Upon staining the major organs with hematoxylin and eosin (H&E), no histological damage was observed as a result of the Ir@WO_3−x_ nanoreactors (Figure S27, Supporting Information). Next, we assessed the anti‐cancer effect of the Ir@WO_3−x_ nanoreactors as dual catalysts in vivo using a triple‐negative breast cancer (TNBC) model. After reaching a tumor volume of ≈100 mm^3^, the mice were randomly divided into six treatment groups. The treatments are illustrated in **Figure** **8**a.

When 1064‐nm NIR‐II laser irradiation was administered to mice that had received Ir@WO_3−x_ nanoreactor injection, the heat at tumor sites increased rapidly (by 20.3 °C in <5 min; Figure 8b,c). For 14 days after therapy, the tumor size was measured every 2 days. As shown in Figure 8d, tumors injected with PBS or Ir@WO_3−x_ nanoreactors alone grew most rapidly. After 14 days of treatment, the tumor volumes reached ≈514.4 and ≈442.6 mm^3^, respectively, which were 3‐ and 5‐fold the initial value. This indicated that nanoreactor treatment without RT was not significantly toxic. Tumor suppression was greater in the Ir@WO_3−x_ nanoreactors + X‐ray irradiation group than in the X‐ray irradiation alone group, highlighting that the nanoreactors enhanced the radiosensitivity of tumor cells due to oxidative damage. The results obtained for the Ir@WO_3−x_ nanoreactors + NIR‐II + X‐ray irradiation group further confirmed that with the alleviation of hypoxia, the radiotherapeutic effect was significantly enhanced with a minimal mean tumor volume of just ≈54.7 mm^3^. Following the euthanasia of the mice, the tumors were collected, measured, and photographed (Figure 8e). Similar to the tumor growth curves, tumor weight showed the most obvious tumor‐inhibiting effect in the Ir@WO_3−x_ nanoreactors + NIR‐II + X‐ray irradiation group, further demonstrating the synergistic advantages of the alleviation of tumor hypoxia and enhanced production of ·OH radicals (Figure S28, Supporting Information). Tumor tissue sections stained with H&E showed that the combined treatment caused the most necrosis and apoptosis; the single therapies caused partial death, nucleus rupture, and tumor cell ablation (Figure 8f). Next, we determined the biosafety of the Ir@WO_3−x_ nanoreactors when used as radiosensitizers. All the mice maintained good health, with negligible fluctuations in body weight during the 14‐day period. The average body weights of mice in the control and Ir@WO_3−x_ nanoreactors + NIR‐II + X‐ray irradiation group reached an increase in body weights of 7.62% and 12.65% compared to the first day, with an overall slight growth trend (Figure 8g), which indicated that the Ir@WO_3−x_ nanoreactors had no apparent toxicity. H&E‐stained organ sections confirmed the lack of toxicity for all treatments, confirming the safety of Ir@WO_3−x_ nanoreactor use for radiosensitization after various treatments (Figure S29, Supporting Information).

Bioluminescence imaging was used to noninvasively monitor post‐treatment tumor proliferation and metastasis in vivo (Figure 8h; Figure S30, Supporting Information). The Ir@WO_3−x_ nanoreactors effectively inhibited the growth of TNBC when combined with X‐ray and/or NIR‐II irradiation, with the most significant inhibitory effects (tumor volume reduction and lack of metastasis) observed in the Ir@WO_3−x_ nanoreactors + NIR‐II + X‐ray irradiation group. Following treatment, we examined and photographed the mice's India ink‐stained lungs (Figure 8i; Figure S31, Supporting Information). No pulmonary metastasis was observed after treatment with Ir@WO_3−x_ nanoreactors combined with X‐ray and/or NIR‐II irradiation, suggesting that combined treatment successfully inhibited tumor metastasis.

Subsequently, tumor section staining confirmed the combined sensitizing effect of Ir@WO_3−x_ nanoreactors, which reduced hypoxia and enhanced ·OH bursts. Immunohistochemical staining showed radioenhancement mediated by the Ir@WO_3−x_ nanoreactors. Coinciding with the in vitro results and confirming the alleviation of tumor hypoxia, HIF‐1α‐positive signals were reduced in tumors in the Ir@WO_3−x_ nanoreactors + NIR‐II irradiation group (Figure 8j,k). The in vivo results for O_2_ production and DNA damage caused by the combined treatment also coincided with the in vitro results (Figure 8l,m). The Ki‐67 results for the Ir@WO_3−x_ nanoreactors + NIR‐II + X‐ray irradiation group reflected a significant reduction of tumor proliferation (Figure 8n,o). Overall, these outcomes reflected the superior biological compatibility and potency of anticancer treatment involving Ir@WO_3−x_ nanoreactors.

## 3 Conclusion

In this study, we engineered and evaluated Ir@WO_3−x_ nanoreactors as a novel nanoplatform for precise CT image‐guided radiosensitization, optimizing anticancer RT. Leveraging their POD‐like activity, the Ir@WO_3−x_ nanoreactors respond to endogenous overexpressed H_2_O_2_ in the TME during PTT and RT, facilitating oxidation reactions that generate highly toxic ·OH. In contrast, ·OH production is limited in normal cells due to H_2_O_2_ deficiency. Thus, the Ir@WO_3−x_ nanocatalysts effectively achieve tumor‐specific radiation enhancement. In addition, Ir@WO_3−x_ exhibits strong X‐ray absorption, increasing radiation doses to tumor regions and inducing ROS production. Their high photothermal conversion efficiencies enable direct cancer cell killing via elevated temperatures and ROS generation, making them excellent agents for multimodal therapy. Moreover, the Ir@WO_3−x_ nanoreactors exhibit CAT‐like properties under NIR‐II irradiation, triggering the release of O_2_ from H_2_O_2_ decomposition and thereby further reversing the hypoxic TME and again enhancing the sensitivity of RT. This dual radiosensitization effect maximizes the radiosensitizing effect of radiotherapy and greatly improves the anti‐tumor efficacy of RT. The dual‐catalytic synergistic therapy achieved with the Ir@WO_3−x_ nanoreactors also minimizes damage to normal tissues. At the same time, Ir@WO_3−x_ nanoreactors can serve as CT contrast agents to facilitate precision anti‐tumor RT, representing an innovative direction for multimodal cancer treatment.

## 4 Experimental Section

### Materials

Tungsten hexachloride (WCl_6_) and isopropyl alcohol (C_3_H_7_OH) were purchased from Innochem (Beijing, China). Ascorbic acid (AA) and sodium hexachloroiridate (III) hydrate (Na_3_IrCl_6_·xH_2_O) were purchased from Sigma‐Aldrich. The China National Pharmaceutical Group Corporation (Beijing, China) provided the sodium borohydride (NaBH_4_). Anaerobic gas production bags (2.5 mL) were purchased from Hope Bio‐technology Co., Ltd. (Qingdao, China). Fetal bovine serum (FBS), RPMI‐1640 medium, and Dulbecco's modified Eagle medium (DMEM) were acquired from Thermo Fisher Scientific Inc (Shanghai, China). Beyotime Biotechnology (Shanghai, China) supplied Hoechst 33342; 2′,7′‐dichlorodihydrofluorescein diacetate (DCFH‐DA), comet assay kits, DNA damage [phospho‐histone H2AX (γ‐H2AX) immunofluorescence], mitochondrial membrane potential (JC‐1) assay kits, annexin V–fluorescein isothiocyanate (FITC) apoptosis detection kit, and calmodulin AM‐propidium iodide. A cell counting kit‐8 (CCK‐8), hypoxia‐inducible factor‐1α (HIF‐1α), γ‐H2AX, and Ki‐67 were obtained from Bioss Biotechnology Co., Ltd. (Shanghai, China). Beijing Vital River Laboratory Animal Technology Co., Ltd. (Beijing, China) provided the BALB/c female mice (age 6–8 weeks, weight 20 g).

### Characterization of Ir@WO_3−x_ Nanoreactors

The Ir@WO_3−x_ nanoreactors' size and morphology were examined using transmission electron microscopy (TEM; Leo 922; Zeiss). WO_3−x_ was fractured by an ultrasonic fragmenter (SONICS VCX130; SONICS & MATERIALS, Inc.). The crystal phase of the samples was determined by X‐ray diffraction (XRD; XD‐D1; Shimadzu). The hydrodynamic particle size and surface charge status of Ir@WO_3−x_ nanospheres were determined by zeta potential measurement (Brookhaven Instruments). The binding energies of W 4f and Ir 4f were confirmed using X‐ray photoelectron spectroscopy (XPS; PHI 5600; PerkinElmer). The optical properties of the samples were determined using a spectrophotometer (U‐4100; Hitachi). The temperature elevation caused by NIR laser irradiation was observed by thermographic analysis (FLIR System i7; FLIR Systems Inc.). Tumor metastasis was monitored using a small animal live imaging system (IVISL Lumina III; PerkinElmer). Computed tomography was conducted with a device from Platinum Elmer Trading Co., Ltd. (Shanxi, China). Flow cytometry (Becton Dickinson) was used to detect cell apoptosis.

### Synthesis of Ir@WO_3−x_ Nanoreactors

At room temperature, 100 mg WCl_6_ was dissolved in 50 mL n‐propyl alcohol and stirred until completely dissolved. A 100‐ml PTFE‐lined high‐pressure sterilizer was used to heat the mixture for 20 h at 200 °C. The reactor cooled spontaneously following the reaction. WO_3−x_ NRs were obtained by centrifugation and washed. The WO_3−x_ NRs were then fractured by an ultrasonic fragmenter at 65 W power for 15 h. The fractured WO_3−x_ NRs were dried under vacuum at 60 °C overnight. Subsequently, 50 mL 18.2‐MU*cm ultrapure water was heated to 95 °C in a round‐bottomed flask, and 50 mg AA and 10 mg Na_3_IrCl_6_·xH_2_O were added. The previously fragmented WO_3−x_ NRs were introduced into this solution and stirred thoroughly for 15 h. After 15 h, the mixture was centrifuged at 10 000 rpm for 5 min. The supernatant was poured off, and the precipitate was retained. Then, 1 mL of water was added to the precipitate and dispersed well under ultrasound. The mixture was then added with 25 mg of NaBH_4_ dissolved in 1 mL of ice water. It was stirred vigorously for 15 min. Finally, the solution was dried under a vacuum at 60 °C overnight.

### Evaluation of the Photothermal Effect of Ir@WO_3−x_

Aqueous solutions (500 µL each) of Ir@WO_3−x_ at various concentrations were exposed to 808‐ and 1064‐nm laser irradiation (1 W·cm^−2^) for 10 min. Using a thermal imager to measure temperatures in real‐time. An aqueous solution (500 µL) of Ir@WO_3−x_ at the same concentration was then irradiated with a 1064 nm laser at different power levels. Real‐time monitoring was performed on the temperature. In order to evaluate the thermal stability of Ir@WO_3−x_, 500 µL of aqueous solution (0.25 mg mL^−1^) was irradiated with a 1064 nm NIR‐II laser (1 W·cm^−2^) for five on–off cycles, and real‐time temperature measurements were recorded.

### Measurement of POD‐like Activity

Acetate buffer solution (180 µL) and Ir@WO_3−x_ solution (10 µL, 2 mg mL^−1^) were added sequentially to a 96‐well plate to achieve a total volume of 200 µL. Different concentrations of H_2_O_2_ and tetramethylbenzidine (TMB; 5 µL each) were then added to the wells. A microplate reader was used to measure the absorbance at 652 nm. In this system, the TMB concentration was 25 mm and the final H_2_O_2_ concentration gradient was 0, 0.01, 0.05, 0.1, 0.7, and 1 mm. Repeated the experiment three times.

### ROS Analysis

Hazardous extracellular ROS were identified using a DCFH‐DA fluorescent probe. DCFH‐DA was not fluorescent but produces fluorescent DCF when it reacts with ROS. DCFH‐DA (12.5 µL) was added to 0.5 mL dimethyl sulfoxide (DMSO), and the resulting solution was reacted with 2 mL 0.01‐M NaOH solution for 0.5 h. To stop the reaction and obtain the ROS detection solution, 10 mL PBS (10 mm, pH 6.5) was added. Ir@WO_3−x_ (0.25 mg mL^−1^), WO_3−x_ (0.25 mg mL^−1^), and H_2_O_2_ (0.1 mm) were mixed separately with the detection solution. The fluorescence of DCF (Ex: 488 nm; Em: 525 nm) produced by DCFH‐DA under ROS stimulation was then detected.

### Detection of Hydroxyl Radicals

To verify the nanomaterial's POD‐like catalytic activity, the ·OH‐specific indicator TA was used to analyze the ·OH production induced by NIR/X‐ray irradiation of the Ir@WO_3−x_ solution. The strong reaction between TA and ·OH forms TAOH, which exhibits fluorescence (Ex: 315 nm; Em: 435 nm). WO_3−x_ and Ir@WO_3−x_ were dissolved in PBS (pH 6.5) to prepare solutions of the same concentration. The samples were irradiated with NIR‐II/X‐ray after adding TA/H_2_O_2_. The liquid was aspirated and centrifuged, and the change in fluorescence intensity was recorded.

### CAT‐like Activity Assay

The O_2_ production capacities of WO_3−x_ (0.1 mg mL^−1^) and Ir@WO_3−x_ (0.1 mg mL^−1^) incubated with H_2_O_2_ solution (0.1 mm) were examined using a portable dissolved O_2_ meter to further evaluate their CAT‐like activity.

### Cell Culture

The Chinese Academy of Sciences (Shanghai, China) cell bank provided the 4T1 mouse breast cancer cells and the HeLa human cervical adenocarcinoma cells. Firefly luciferase‐expressing 4T1 cancer cells were purchased from Cobioer (Nanjing, China). Cell culture medium using RPMI 1640 medium or DMEM. In a humidified incubator, normoxic cells were cultured at 37 °C and 5% CO_2_. Hypoxic cells that had attached to the wall were cultured further in a box with a hypoxic bag at 37 °C.

### Examination of 4T1 Cellular Uptake

Incubation with Ir@WO_3−x_ nanoreactors for 24 h was conducted on 4T1 cells. They were then trypsin digested and centrifuged, followed by overnight fixation. The next day, the samples were prefixed with 2.5% glutaraldehyde for 1 h and washed with 0.1 M phosphate buffer (pH 7.2). A 1% osmic acid fixative was applied, followed by rinsing. Samples were dehydrated and stored in a refrigerator at 4 °C. They were soaked overnight in different concentrations of pure acetone and embedding solution, followed by embedding. After 5 days of polymerization, the samples were sectioned using an ultramicrotome, stained, and observed and photographed under an electron microscope.

### Examination of HeLa Cellular Uptake

HeLa cells that had grown several times were plated into culture flasks, and a complete culture medium containing Ir@WO_3−x_ nanoreactors was added and incubated for 6, 12, and 24 h, respectively. After digesting and centrifuging the cells, the supernatant was discarded and the pellet was transferred to an Erlenmeyer flask. Concentrated nitric acid was added and the samples were heated until the liquid was nearly completely evaporated. Ultrapure water was added and the procedure was repeated until the liquid was transparent. The volume of 2% nitric acid solution was adjusted to 5 mL. The Ir and W contents were measured using ICP‐OES.

### Cytotoxicity Assays

The cytotoxicity of Ir@WO_3−x_ against 4T1 cells, HeLa cells, and HUVEC cells in vitro was evaluated using the CCK‐8 method. In 96‐well plates, the cells were grown at a density of 5 × 10^3^ cells per well for 24 h. Hypoxic cells were cultured further in an incubator with a hypoxic bag at 37 °C. A variety of concentrations of Ir@WO_3−x_ was used in the wells to replace the medium. For RT experiments, different doses of X‐ray irradiation (2, 4, 6, 8, and 10 Gy) were applied to cells. For synergistic NIR‐II + RT cell viability determination, 1064‐nm NIR‐II laser irradiation (1 W·cm^−2^) was applied for 5 min, and X‐ray irradiation was applied 1 h later. To assess cell viability, 10 µL CCK‐8 solution was applied to each well and incubated for 1 h at 37 °C. Using a microplate reader (Infinite M200; Tecan) the absorbance of the samples was measured at 450 nm. Six replicates per group were examined.

### Intracellular O_2_ Assay

The hypoxia probe [Ru(dpp)_3_]Cl_2_ was used to determine the levels of intracellular O_2_ (which quenches red fluorescence). 4T1 and HeLa cells were seeded into culture wells for 1 day, then incubated with [Ru(dpp)_3_]Cl_2_ (10 µg mL^−1^) for 4 h. After Ir@WO_3−x_ treatment for 12 h, cells were irradiated with a 1064 nm laser (1 W·cm^−2^) for 5 min and incubated with DAPI for 5 min. Images were captured using fluorescence microscopy.

### Detection of Intracellular ROS

DCFH‐DA probes were used to measure ROS production in cells. The ability of X‐ray irradiation to create ROS was assessed by performing fluorescence spectroscopy on a sample containing nanoreactors and DCFH, which was proportionate to the ROS yield.^[^ ^38^ ^]^ 4T1 and HeLa cells were cultured for 1 day and then treated with Ir@WO_3−x_ for 12 h. Next, X‐ray or 1064‐nm NIR‐II irradiation was subjected to the cells. Diluted DCFH‐DA with serum‐free medium (1 mL per well) was added to the cells, which were then incubated in a 37 °C cell incubator for 20 min. Using fluorescence microscopy, the fluorescence intensity of the cells was quantified to represent the production of ROS.

### Intracellular HIF‐1α Analysis

4T1 cells were seeded into six‐well plates fitted with slides, and then Ir@WO_3−x_ was added and incubated for 12 h. After that, a 1064‐nm laser (1 W·cm^−2^) was used to irradiate one treatment group for 5 min. After overnight incubation with HIF‐1α antibodies (bs‐20399R), the cells were treated with secondary antibodies at room temperature for 1 h. Using confocal laser scanning microscopy (CLSM), fluorescence images were captured.

### Western Blot Analysis

Western blot analysis was used to determine the levels of HIF‐1α in the cells. Following the lysis of 4T1 cells, the protein content was quantified and maintained at 30 µg per sample. The samples were separated by sodium dodecyl sulfate‐polyacrylamide gel electrophoresis for 90 min, and the proteins were transferred to a polyvinyl difluoride (PVDF) membrane. After blocking overnight, the membrane was incubated with an HIF‐1α antibody at 4 °C overnight, and treated with the secondary antibody at room temperature for 1 h. A chemiluminescence imaging system (4600SF; Tanon, Shanghai, China) was used to view the bands on the PVDF membranes.

### Colony Formation Assay

Cells were cultured in cell culture plates for 24 h, then nanoreactors were added and cultured for an additional 24 h. For RT experiments, cells were X‐ray irradiated (6 MeV, 6 Gy). For PTT experiments, cells were irradiated with a 1064 nm laser (1 W·cm^−2^) for 5 min. For combined experiments, NIR‐II laser irradiation was applied for 1 h, followed by X‐ray irradiation. After treatment, the cell culture was continued for 14 days. Changing the medium of culture every other day. After fixing and staining the cells, they were rinsed. Cell colonies were photographed and counted using Image J software.

### Examination of Double‐strand DNA Breakage

Immunofluorescence staining with phospho‐histone H2AX (Ser139) antibody was used to detect DNA damage in cells. 4T1 and HeLa cells were cultured for 1 day. They were then incubated with Ir@WO_3−x_ for 12 h and subjected to different treatments. Thereafter, the cells were incubated overnight at 4 °C with γ‐H2AX antibody and then incubated for another 1 h at room temperature with the corresponding secondary antibody. The nuclei were stained with DAPI and subsequently visualized by CLSM.

### Alkaline Comet Assay

Cultured 4T1 cells and HeLa cells with good growth status were incubated with Ir@WO_3−x_ for 12 h, then subjected to NIR‐II laser or X‐ray irradiation. After a single wash in ice‐cold PBS, the cells were centrifuged and reconstituted to a density of 1 × 10^5^ mL^−1^. The cell resuspensions (30 µL each) were then blended with low‐melting‐point agarose (LMA; 70 µL, 37 °C) using the sandwich method to form middle layers. The upper and lower layers were formed with normal‐melting‐point agarose and LMA, respectively. Then electrophoresis was performed in electrophoresis buffer for 25 min. After 5 min of PI solution staining, the cells were photographed under a fluorescence microscope.

### Intracellular Live/Dead Cell Detection

Cells were seeded in wells at a density of ≈2 × 10^5^ per well and cultured for 1 day. Ir@WO_3−x_ was added, followed by 12 h additional culture. The treatments were applied to sample groups. The samples were then incubated with AM/PI for 15 min. An inverted fluorescence microscope was used to observe the fluorescence distributions reflecting the live–dead cell ratio.

### Mitochondrial Membrane Potential Assay

Cells were seeded in wells and cultured for 1 day to achieve adhesion. Medium containing Ir@WO_3−x_ was added and incubated for 12 h. NIR (1064 nm, 1 W·cm^−2^) or X‐ray (6 Gy) irradiation was applied for 5 min. JC‐1 was applied to the cells at 37 °C for 20 min, followed by washing and viewing under a fluorescence microscope.

### Flow Cytometry to Detect Cell Apoptosis

Medium containing Ir@WO_3−x_ was added to adherent 4T1 and HeLa cells, which were then incubated for 12 h and subjected to different treatments. After 12 h, the culture solution was pipetted into the prepared corresponding centrifuge tubes. To collect the cells, the samples were centrifuged after being digested with EDTA‐free trypsin. Added Annexin V‐FITC conjugate and resuspended the cells. PI staining solution or Annexin V‐FITC was added, and flow cytometry assays were performed 1 h later.

### Establishment of Mouse Tumor Models

All the experimental procedures involving animals were conducted following a protocol approved by the Harbin Institute of Technology Ethics Committee (IACUC‐2022036). We purchased female BALB/c mice that were 5 weeks old and weighed between 18 and 22 grams from Vitalriver Experimental Animal Technical Co., LTD (Beijing, China). To establish a transplanted tumor model, a 100‐µL subcutaneous injection containing ≈1 million fireflies luciferase‐expressing 4T1 cells was administered to the left back of each mouse. Upon the tumor's volume reaching ≈100 mm^3^, treatment commenced. To establish an orthotopically transplanted tumor model, each mouse's mammary fat pad was incised and injected subcutaneously with a 100‐µL volume containing 1 × 10^4^ firefly luciferase‐expressing 4T1 cells. Tumor‐bearing mice were obtained on the 5^th^ day after suturing. Orthotopically transplanted breast cancers in untreated mice metastasized as they continued to grow.

### Blood Hematology and Biochemical Analysis

Mice were injected with the nanomaterials through the tail vein. Subsequently, at specific time intervals (3 h, 6 h, 12 h, 24 h, 3 d, 7 d), 0.5 mL of blood was taken from the medial canthus vein. A blood analyzer was used for testing, and the pre‐dilution mode was selected to dilute the blood. The indicators detected included white and red blood cells, platelet counts, and hematocrit.

### CT

A 90‐kV, 160‐µA quantum GX micro‐CT imaging system (PerkinElmer, Waltham, MA, USA) was used to assess the in‐vitro and in‐vivo diagnostic potential of Ir@WO_3−x_ as a CT contrast agent. To examine the linearity of the relationship between CT signals and Ir@WO_3−x_ concentrations, different concentrations of Ir@WO_3−x_ (0.3125‐15 mg mL^−1^) were diluted in centrifuge tubes. The tubes were subjected to in‐vitro CT signal detection in order of concentration from small to large. 4T1 mice with metastatic tumors received an intratumorally injected dose of 100 µL of Ir@WO_3−x_ solution (0.1 mg mL^−1^). The mice were then anesthetized in a small‐animal gas anesthesia system and placed into the CT system to select different time points for detection.

### In vivo RT and PTT for Cancer

When the orthotopically transplanted tumors in the mice had reached a volume of ≈100 mm^3^, around the 6th day, the mice were randomly allocated to six treatment groups of five mice each: 1) PBS, 2) Ir@WO_3−x_ (5 mg·kg^−1^), 3) PBS + X‐ray irradiation (6 Gy), 4) Ir@WO_3−x_ (5 mg·kg^−1^) + X‐ray irradiation (6 Gy), 5) Ir@WO_3−x_ (5 mg·kg^−1^)+ NIR‐II irradiation (1064 nm, 1 W·cm^−2^, 5 min), and 6) Ir@WO_3−x_ (5 mg·kg^−1^) + NIR‐II irradiation (1064 nm, 1 W·cm^−2^, 5 min) + X‐ray irradiation (6 Gy). The treatments were administered 12 h after Ir@WO_3−x_ injection. Ir@WO_3−x_ injection and radiation therapy (6 Gy) were performed every other day. From the beginning of treatment, daily measurements were taken of the mice's weight and tumor volume. The relative tumor volume was calculated as volume (mm^3^) = (length × width^2^)/2.

### Biofluorescence Imaging of Tumor Metastasis

Twenty‐eight days after orthotopically implanted tumor treatment, imaging was performed to track metastasis to distant organs. D‐luciferin potassium salt was diluted with Dulbecco's PBS to prepare a working solution (15 mg mL^−1^) and filtered using a 0.2‐µm filter. The filtered working solution (10 µL g^−1^) was injected intraperitoneally into mice inoculated with 4T1‐Luc cells. Ten minutes later, the mice were anesthetized using a gas anesthesia system, and imaging analysis was performed with a small‐animal in vivo imaging system.

### Observation of Lung Nodules

The mouse tracheas were filled with India ink to stain lung tumors, and the tracheas were ligated with surgical sutures to prevent the ink from flowing out. Decolorizing was then performed with Fekete's solution (containing 20 mL formaldehyde, 10 mL glacial acetic acid, 140 mL absolute ethanol, and 30 mL water). After a few minutes, metastatic tumor nodules appeared as white dots on the black lungs. Each group's total number of lung tumor nodules was noted.

### Histological Analyses

Following treatment, the mice were sacrificed and the hearts, livers, spleens, lungs, kidneys, and tumors were removed and fixed in 4% paraformaldehyde, then embedded, sectioned, and dehydrated. The samples were stained with aqueous hematoxylin solution and rinsed under running water. To restore the blue staining, dilute ammonia water was used after differentiation in hydrochloric acid ethanol solution. Following 15 min of soaking in running water, the samples were dyed for 2 min with eosin. They were then rinsed and placed in distilled water. The samples were dehydrated in 70% and 90% alcohol for 10 min, and xylene was used to make the samples transparent. Afterward, the samples were sealed and viewed under the microscope.

### Statistical Analysis

All statistical data were expressed as mean ±SD for at least three independent experiments. The comparison of data between the two groups was analyzed by GraphPad Prism (8.0) using a two‐tailed Student's t‐test. *p < 0.05, **p < 0.01, ***p < 0.001, and ****p < 0.0001 indicated statistically significant differences.

## Conflict of Interest

The authors declare no conflict of interest.

## Supporting information

Supporting Information

## Data Availability

The data that support the findings of this study are available from the corresponding author upon reasonable request.

## References

[1] a) Q. Chen , J. Chen , Z. Yang , J. Xu , L. Xu , C. Liang , X. Han , Z. Liu , Adv. Mater. 2019, 31, 1802228; 10.1002/adma.201802228 30663118

[2] a) W. Jiang , Q. Li , L. Xiao , J. Dou , Y. Liu , W. Yu , Y. Ma , X. Li , Y. You , Z. Tong , H. Liu , H. Liang , L. Lu , X. Xu , Y. Yao , G. Zhang , Y. Wang , J. Wang , ACS Nano 2018, 12, 5684; 29812909 10.1021/acsnano.8b01508

[3] a) E. Rankin , A. Giaccia , Science 2016, 352, 175; 27124451 10.1126/science.aaf4405 PMC4898055

[4] a) T. Anani , S. Rahmati , N. Sultana , A. David , Theranostics 2021, 11, 579; 33391494 10.7150/thno.48811 PMC7738852

[5] a) J. Xie , L. Gong , S. Zhu , Y. Yong , Z. Gu , Y. Zhao , Adv. Mater. 2019, 31,, 1802244; 10.1002/adma.201802244 30156333

[6] N. Zheng , S. Zhang , L. Wang , Z. Qi , Q. Peng , L. Jian , Y. Bai , Y. Feng , J. Shen , R. Wang , J. Jiao , W. Xu , S. Liu , Nano Res. 2021, 15, 2315.

[7] a) B. Hu , X. Xiao , P. Chen , J. Qian , G. Yuan , Y. Ye , L. Zeng , S. Zhong , X. Wang , X. Qin , Y. Yang , Y. Pan , Y. Zhang , Biomaterials 2022, 290, 121811; 36201948 10.1016/j.biomaterials.2022.121811

[8] a) X. Zhang , X. Chen , Y. Zhao , Nano‐Micro Lett. 2022, 14, 95; 10.1007/s40820-022-00828-2 PMC8986955 35384520

[9] a) K. de Visser , J. Joyce , Cancer Cell 2023, 41, 374; 36917948 10.1016/j.ccell.2023.02.016

[10] G. Xi , S. Ouyang , P. Li , J. Ye , Q. Ma , N. Su , H. Bai , C. Wang , Angew. Chem. Int. Ed. 2012, 51, 2395. 10.1002/anie.201107681 22282345

[11] L. Feng , Z. Dong , C. Liang , M. Chen , D. Tao , L. Cheng , K. Yang , Z. Liu , Biomaterials 2018, 181, 81. 30077139 10.1016/j.biomaterials.2018.07.049

[12] S. He , X. Jia , S. Feng , J. Hu , Small 2023, 19, e2300078. 37226364 10.1002/smll.202300078

[13] D. Xu , L. Wu , H. Yao , L. Zhao , Small 2022, 18, e2203400. 35971168 10.1002/smll.202203400

[14] W. Fu , X. Zhang , L. Mei , R. Zhou , W. Yin , Q. Wang , Z. Gu , Y. Zhao , ACS Nano 2020, 14, 10001. 32658453 10.1021/acsnano.0c03094

[15] L. Wen , L. Chen , S. Zheng , J. Zeng , G. Duan , Y. Wang , G. Wang , Z. Chai , Z. Li , M. Gao , Adv. Mater. 2016, 28, 5072. 27136070 10.1002/adma.201506428

[16] Q. Lv , J. Tan , Z. Wang , P. Gu , H. Liu , L. Yu , Y. Wei , L. Gan , B. Liu , J. Li , F. Kang , H. Cheng , Q. Xiong , R. Lv , Nat. Commun. 2023, 14, 2717. 37169769 10.1038/s41467-023-38198-x PMC10175504

[17] Y. Xuan , X. Q. Yang , Z. Y. Song , R. Y. Zhang , D. H. Zhao , X. L. Hou , X. L. Song , B. Liu , Y. D. Zhao , W. Chen , Adv. Funct. Mater. 2019, 29, 1900017.

[18] Z. Du , X. Wang , X. Zhang , Z. Gu , X. Fu , S. Gan , T. Fu , S. Xie , W. Tan , Angew. Chem. Int. Ed. 2023, 62, e202302525. 10.1002/anie.202302525 36930411

[19] Z. Wang , M. Wang , X. Wang , Z. Hao , S. Han , T. Wang , H. Zhang , Biosens. Bioelectron. 2023, 220, 114883. 36395731 10.1016/j.bios.2022.114883

[20] a) W. Liu , H. Bai , X. Li , W. Li , J. Zhai , J. Li , G. Xi , J. Phys. Chem. Lett. 2018, 9, 4096; 29979872 10.1021/acs.jpclett.8b01624

[21] L. Yang , Z. Wenyao , W. Yinghui , L. Jianhua , J. Longhai , Z. Tianqi , Z. Songtao , Z. Ying , S. Shuyan , L. Chengyu , Z. Junjie , Y. Yang , Z. Hongjie , Angew. Chem. Int. Ed. 2019, 58, 2407.

[22] a) M. Ushio‐Fukai , Y. Nakamura , Cancer Lett 2008, 266, 37; 18406051 10.1016/j.canlet.2008.02.044 PMC2673114

[23] Y. Dai , C. Xu , X. Sun , X. Chen , Chem. Soc. Rev. 2017, 46, 3830. 28516983 10.1039/c6cs00592f PMC5521825

[24] S. Zwiehoff , J. Johny , C. Behrends , A. Landmann , F. Mentzel , C. Bäumer , K. Kröninger , C. Rehbock , B. Timmermann , S. Barcikowski , Small 2022, 18, e2106383. 34921500 10.1002/smll.202106383

[25] S. K. Deb , Sol. Energy Mater. Sol. Cells 2008, 92, 245.

[26] a) H. Peng , P. Liu , D. Lin , Y. Deng , Y. Lei , W. Chen , Y. Chen , X. Lin , X. Xia , A. Liu , Chem. Commun. 2016, 52, 9534; 10.1039/c6cc03245a 27381501

[27] M. Sousa de Almeida , E. Susnik , B. Drasler , P. Taladriz‐Blanco , A. Petri‐Fink , B. Rothen‐Rutishauser , Chem. Soc. Rev. 2021, 50, 5397. 33666625 10.1039/d0cs01127d PMC8111542

[28] a) W. Tang , Z. Yang , L. He , L. Deng , P. Fathi , S. Zhu , L. Li , B. Shen , Z. Wang , O. Jacobson , J. Song , J. Zou , P. Hu , M. Wang , J. Mu , Y. Cheng , Y. Ma , L. Tang , W. Fan , X. Chen , Nat. Commun. 2021, 12, 523; 33483518 10.1038/s41467-020-20860-3 PMC7822893

[29] A. Kumar , V. Goudar , B. Nahak , P. Tsai , H. Lin , F. Tseng , Small 2023, 20, e2307955. 38148312 10.1002/smll.202307955

[30] X. Wang , R. Wu , P. Zhai , Z. Liu , R. Xia , Z. Zhang , X. Qin , C. Li , W. Chen , J. Li , J. Zhang , J Extracell Vesicles 2023, 12, e12310. 36748335 10.1002/jev2.12310 PMC9903130

[31] S. Song , G. Zhang , X. Chen , J. Zheng , X. Liu , Y. Wang , Z. Chen , Y. Wang , Y. Song , Q. Zhou , J. Nanobiotechnol. 2023, 21, 257. 10.1186/s12951-023-02020-z PMC10405507 37550736

[32] a) J. Kim , H. Cho , H. Jeon , D. Kim , C. Song , N. Lee , S. Choi , T. Hyeon , J. Am. Chem. Soc. 2017, 139, 10992; 28737393 10.1021/jacs.7b05559

[33] a) Z. Zhang , M. Lu , C. Chen , X. Tong , Y. Li , K. Yang , H. Lv , J. Xu , L. Qin , Theranostics 2021, 11, 3167; 33537080 10.7150/thno.52028 PMC7847686

[34] a) J. D. Dunn , L. Alvarez , X. Zhang , T. Soldati , Redox Biol. 2015, 6, 472; 26432659 10.1016/j.redox.2015.09.005 PMC4596921

[35] N. Ayoub , A. Jeyasekharan , J. Bernal , A. Venkitaraman , Nature 2008, 453, 682. 18438399 10.1038/nature06875

[36] a) D. Sreerangaraja Urs , W. Wu , K. Komrskova , P. Postlerova , Y. Lin , C. Tzeng , S. Kao , Int. J. Mol. Sci. 2020, 21, 3592; 32438750 10.3390/ijms21103592 PMC7279321

[37] T. Liu , Y. Chao , M. Gao , C. Liang , Q. Chen , G. Song , L. Cheng , Z. Liu , Nano Res. 2016, 9, 3003.

[38] S. Liu , L. Fang , H. Ding , Y. Zhang , W. Li , B. Liu , S. Dong , B. Tian , L. Feng , P. Yang , ACS Nano 2022, 16, 20805. 36378717 10.1021/acsnano.2c08047

