# Correction to “Computed Tomography Imaging Guided Microenvironment‐Responsive Ir@WO_3‐x_ Dual‐Catalytic Nanoreactor for Selective Radiosensitization”

**DOI:** 10.1002/advs.202502913

**Published:** 2025-02-28

Song, J., Feng, Y., Yan, J., Wang, Y., Yan, W., et al. (2024) Computed Tomography Imaging Guided Microenvironment‐Responsive Ir@WO_3‐x_ Dual‐Catalytic Nanoreactor for Selective Radiosensitization. Adv. Sci. 11(38): e2405192.

https://doi.org/10.1002/advs.202405192

In the original article, “The symbol for Yue Feng's co‐first author was missing. And it was not well reflected in the main text.” This should have read: “Jiayu Song and Yue Feng contributed equally to this work.”

The corrected contents are provided as follows: This work was supported by the National Natural Science Foundation of China (No. U20A20339, 82271880, and 82303691), China Postdoctoral Science Foundation (2022TQ0094), Shenzhen Medical Research (A2302046), the Natural Science Foundation of Heilongjiang Province of China for Excellent Young Scholars (No. YQ2022E020), Heilongjiang Touyan Team (HITTY‐20190036) and the Fundamental Research Funds for the Central Universities. Jiayu Song and Yue Feng contributed equally to this work.

We apologize for this error.

