# Supplementary material for: Computed Tomography Imaging Guided Microenvironment‐Responsive Ir@WO3−x Dual‐Catalytic Nanoreactor for Selective Radiosensitization
Source: Adv Sci (Weinh). 2024 Aug 5;11(38):2405192. doi: 10.1002/advs.202405192 (PMC11481196; doi:10.1002/advs.202405192)
Supplement: Supplementary file 1 — Supporting Information [file ADVS-11-2405192-s001.pdf]

ADVANCED SCIENCE
Open Access

Supporting Information

for *Adv. Sci.*, DOI 10.1002/advs.202405192

Computed Tomography Imaging Guided Microenvironment-Responsive Ir@WO_{3-x} Dual-Catalytic Nanoreactor for Selective Radiosensitization

Jiayu Song, Yue Feng, Jiazhuo Yan, Ying Wang, Weixiao Yan, Nan Yang, Tusheng Wu, Sijia Liu, Yuan Wang, Nannan Zheng, Liangcan He* and Yunyan Zhang**

Supporting Information

Computed Tomography Imaging Guided Microenvironment-Responsive $Ir@WO_{3-x}$ Dual-Catalytic Nanoreactor for Selective Radiosensitization

Jiayu Song,[a,b,1] *Yue Feng,*[d,a,1] *Jiazhuo Yan,*[a] *Ying Wang,*[a] *Weixiao Yan,*[b] *Sijia Liu,*[a] *Nan Yang,*[a] *Tusheng Wu,*[a] *Yuan Wang,*[a] *Nannan Zheng* [b,c*] *Liangcan He,*[b,c*] *Yunyan Zhang,*[a*]

[a]Department of Gynecological Radiotherapy, Harbin Medical University Cancer Hospital, Harbin, 150001, China.

[b]School of Medicine and Health, Key Laboratory of Microsystems and Microstructures Manufacturing, Harbin Institute of Technology, Harbin, 150001, China.

[c]Zhengzhou Research Institute, Harbin Institute of Technology, Zhengzhou, Henan, 450000, China.

[d]Department of Gynecological Oncology, Zhejiang Cancer Hospital, Zhejiang，310022，China.

*Corresponding authors: Yunyan Zhang; Liangcan He; Nannan Zheng

Email address: zhangyunyan@hrbmu.edu.cn; liangcanhe@hit.edu.cn;

zhengnannan@hit.edu.cn

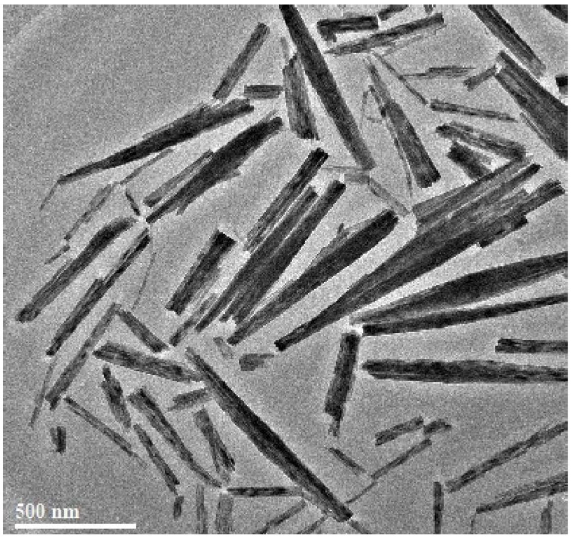

Figure S1. TEM image of WO_{3-x}.

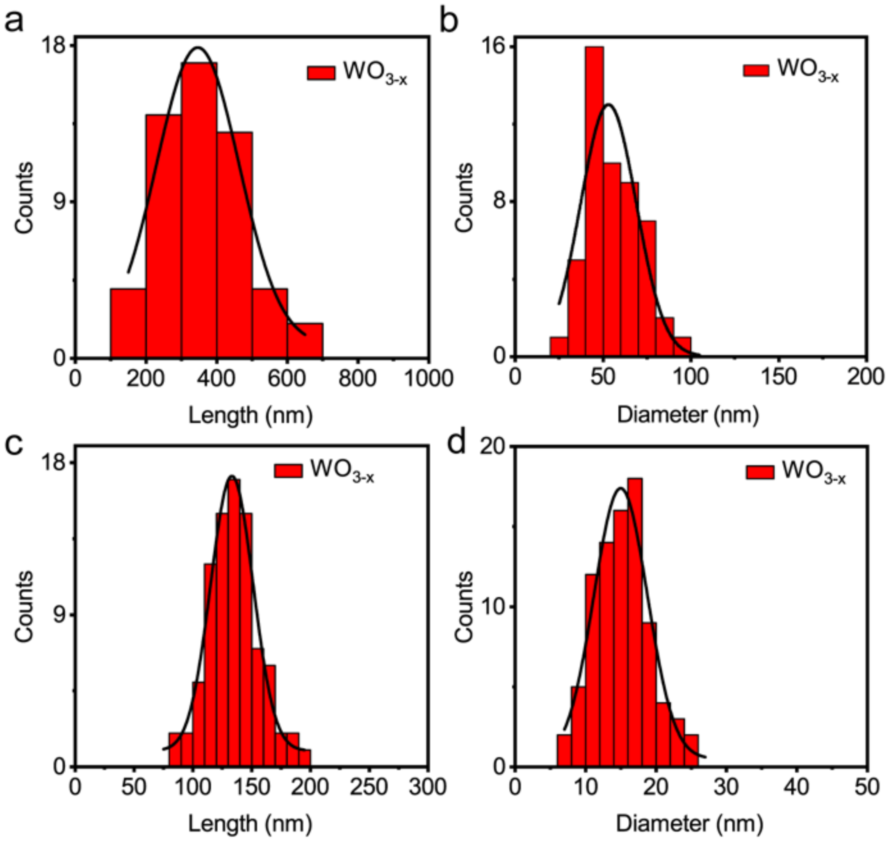

Figure S2. Long- and short-diameter Gaussian distributions of WO_{3-x} NRs (a, b) and

WO_{3-x} NRs after ultrasonic fracture (c, d).

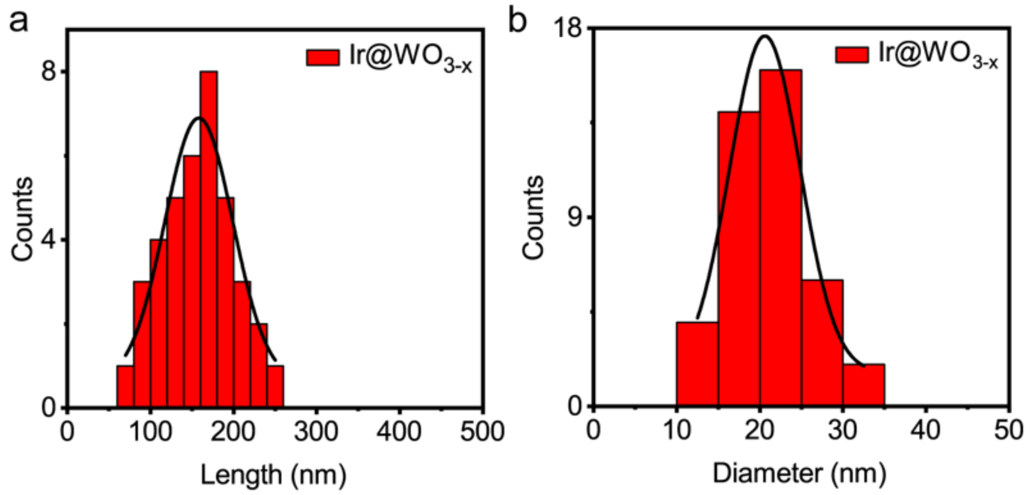

Figure S3. Long- and short-diameter Gaussian distributions of Ir@WO_{3-x} nanoreactors (a, b).

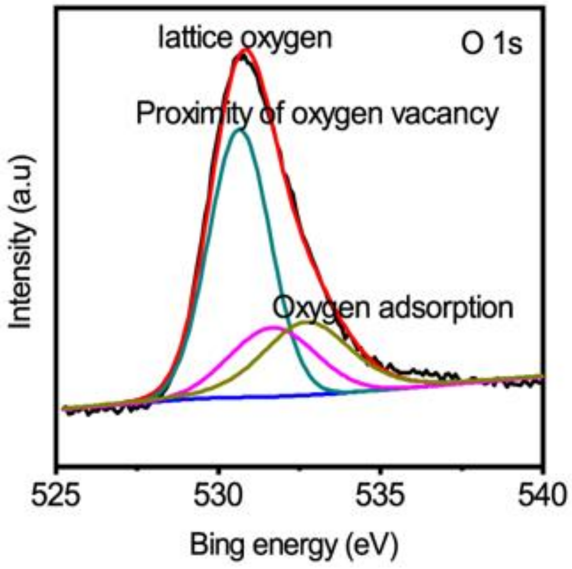

Figure S4. XPS spectra of O 1s or Ir@WO_{3-x} nanoreactors.

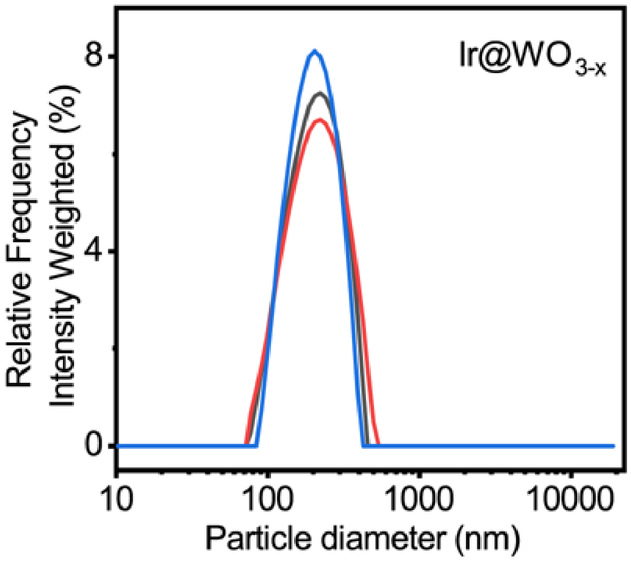

Figure S5. Hydration kinetic diameters of Ir@WO_{3-x} nanoreactors.

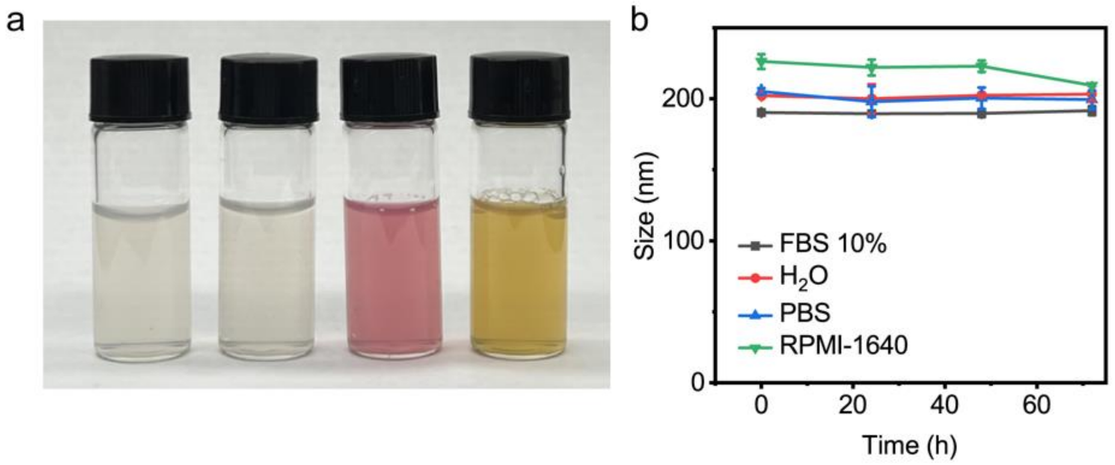

Figure S6. Photograph of Ir@WO_{3-x} dispersed in various solvents (0.25 mg/mL) for 72 h (a). The DLS of Ir@WO_{3-x} in H_2O, PBS, FBS, and RPMI 1640 for 72 h (b).

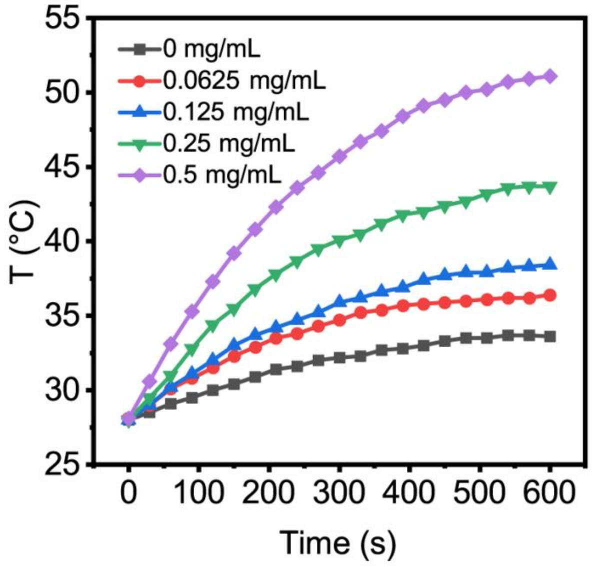

Figure S7. Curves of temperature increase for Ir@WO_{3-x} nanoreactors under 808-nm laser irradiation (1 W·cm^{-2}).

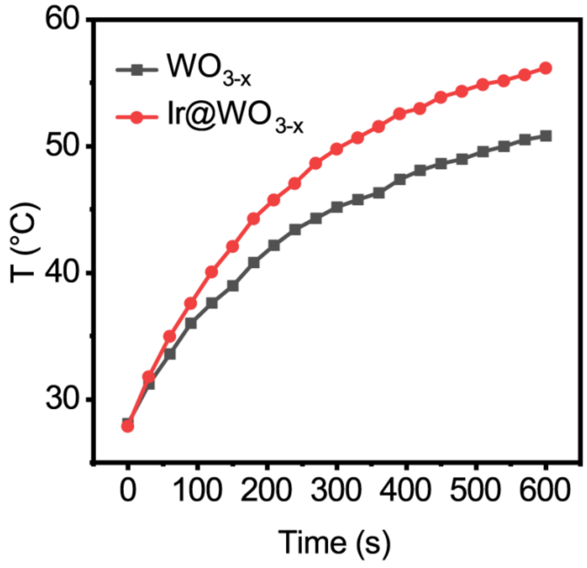

Figure S8. Temperature increase curves for Ir@WO_{3-x} nanoreactors (0.25 mg/mL) and WO_{3-x} NRs (0.25 mg/mL) under 1064-nm laser irradiation (1 W·cm^{-2}).

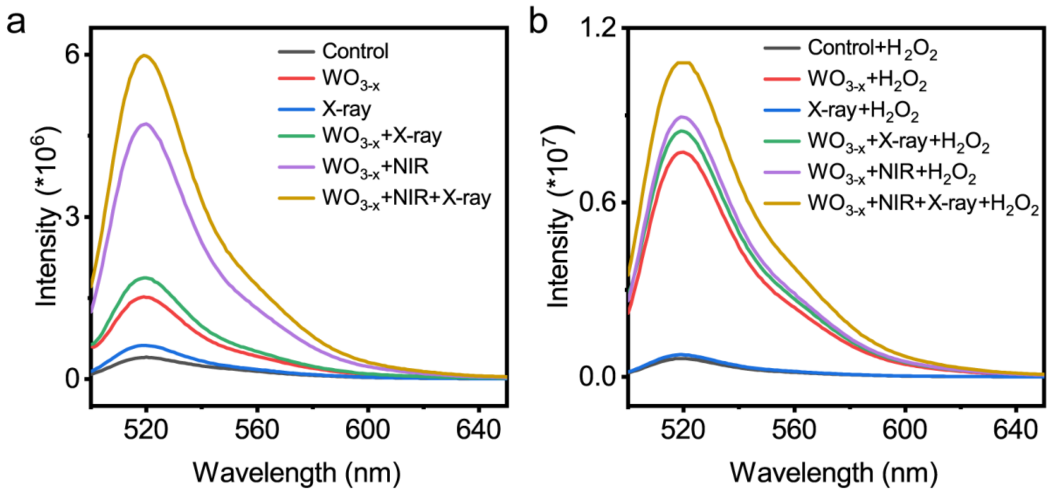

Figure S9. DCFH fluorescence spectra of ROS in the WO_{3-x} NRs (a) and WO_{3-x} NRs + H_2O_2 (b) groups.

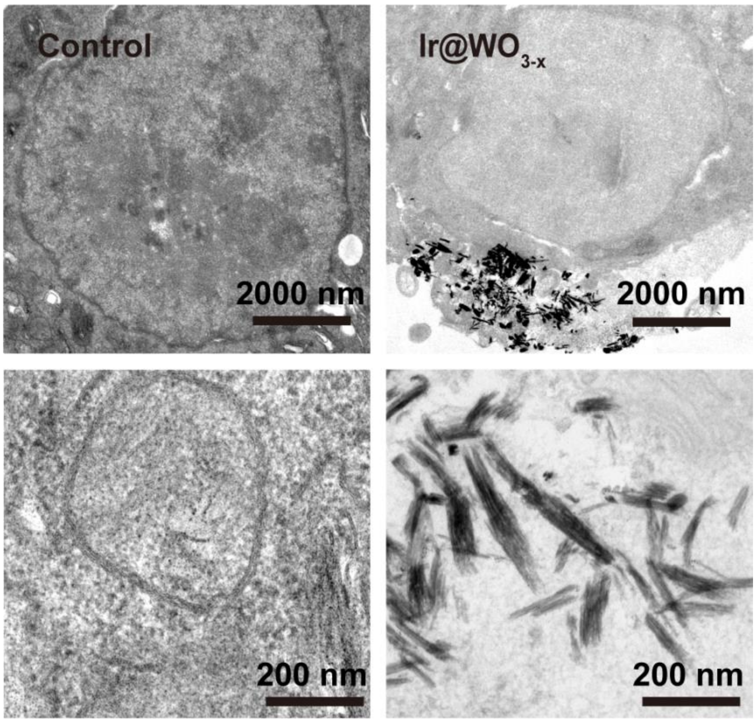

Figure S10. TME images of 4T1 cells treated with Ir@WO_{3-x} for 12 h.

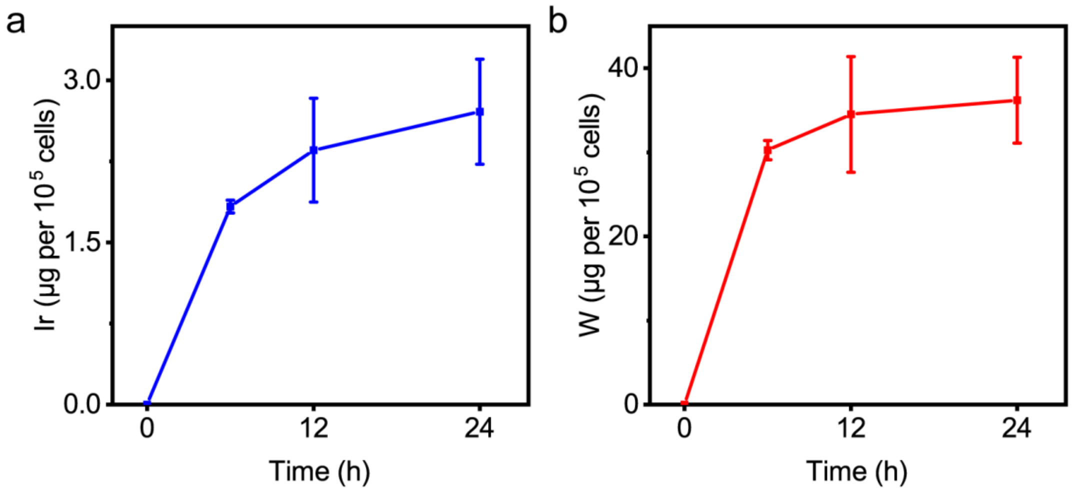

Figure S11. Uptake of Ir and W from Ir@WO_{3-x} nanoreactors by HeLa cells after 12 and 24 h (mean ± SD, n = 3).

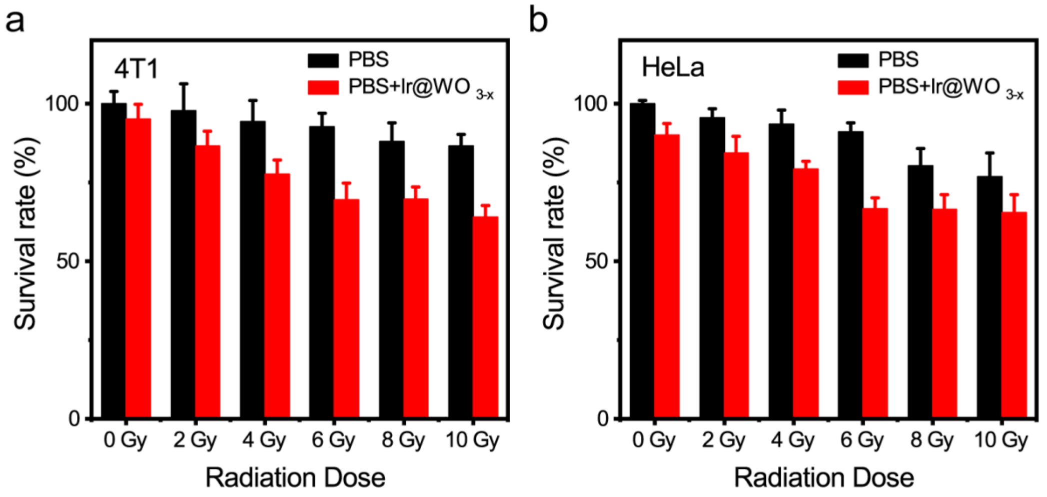

Figure S12. Optimal radiotherapy doses for 4T1 and HeLa cells (mean ± SD, n = 6).

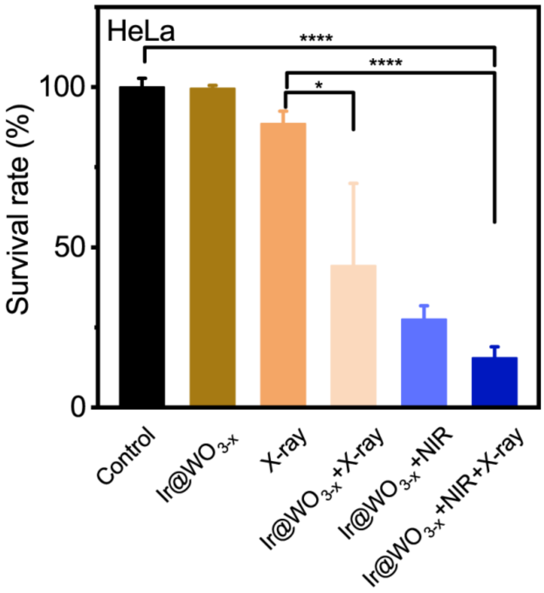

Figure S13. HeLa cell survival after various treatments (mean ± SD, n = 4). P values were assessed using a two-tailed Student's t test with GraphPad Prism 8.0. *P < 0.05, ****P < 0.0001.

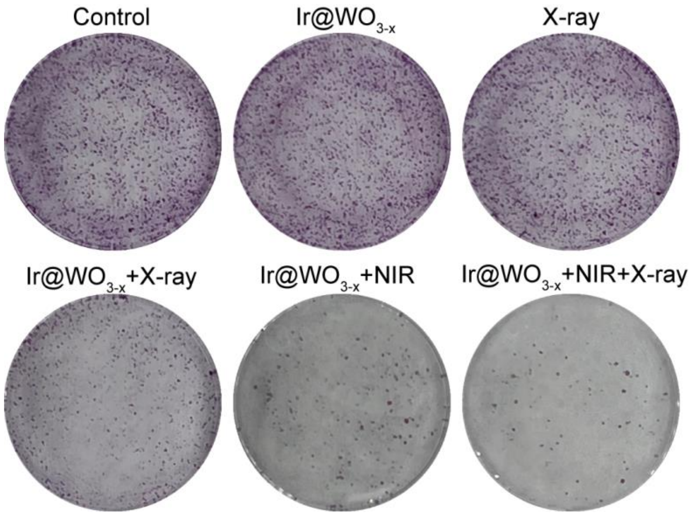

Figure S14. Photographs of HeLa cell colony formation in the various treatment groups.

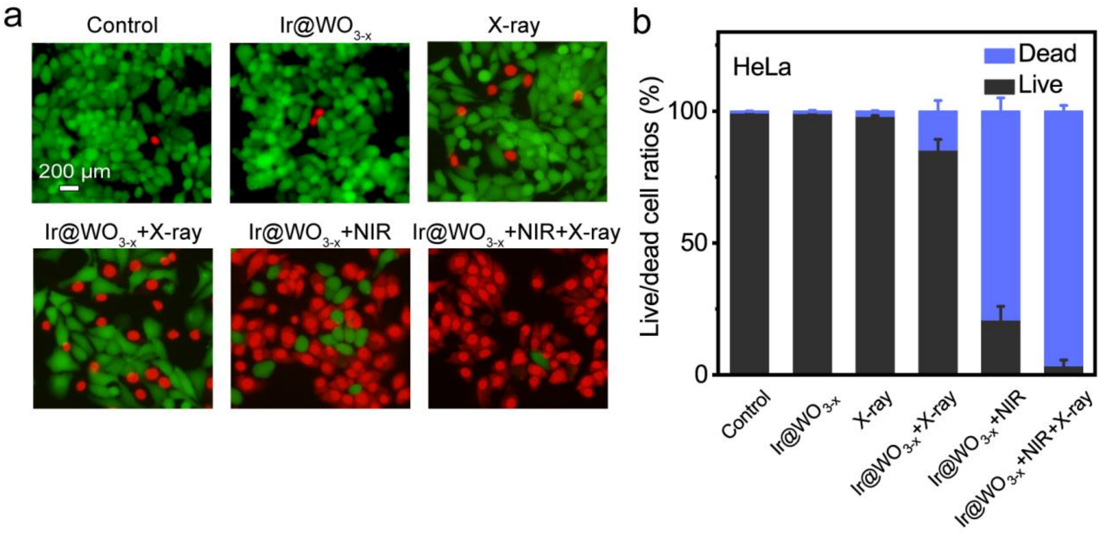

Figure S15. Calcein-AM/PI fluorograms (a) and statistics (b) for HeLa cells in different treatment groups (mean ± SD, n = 3).

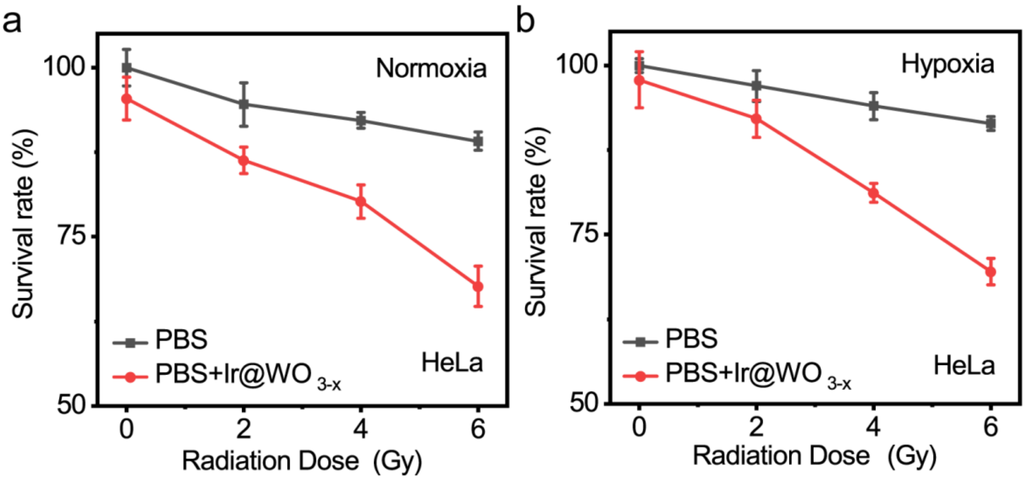

Figure S16. Cytotoxicity in HeLa cells treated with Ir@WO_{3-x} nanoreactors under normoxic (a) and hypoxic (b) conditions, followed by x-ray irradiation (mean ± SD, n = 3).

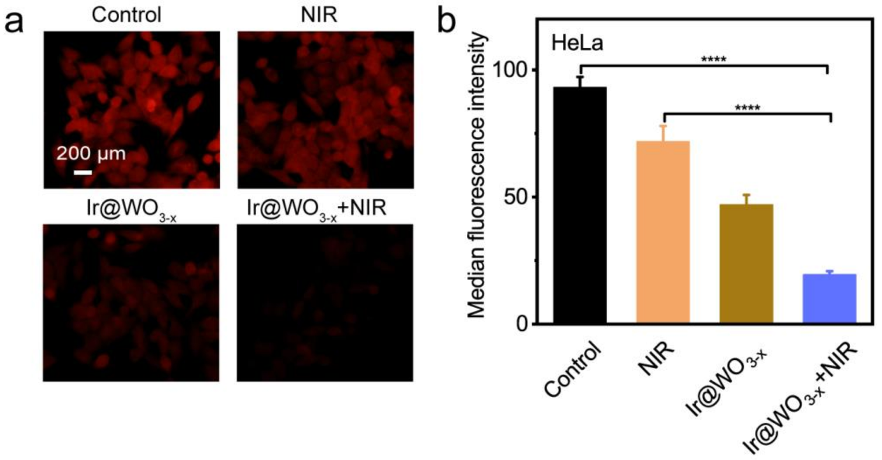

Figure S17. $[Ru(dpp)_3]Cl_2$ fluorescence intensity in HeLa cells in different treatment groups (a) and statistical analysis (b) (mean ± SD, n = 3). P values were assessed using a two-tailed Student's t test with GraphPad Prism 8.0. ****$P < 0.0001$.

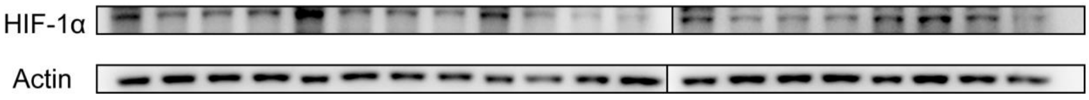

Figure S18. HIF-1α expression levels in 4T1 cells treated with varying $Ir@WO_{3-x}$ nanoreactors concentrations at different time points, detected by western blot analysis. From left to right: control; 0.1 mg/mL, 8 h; 0.2 mg/mL, 8 h; 0.4 mg/mL, 8 h; control; 0.1 mg/mL, 12 h; 0.2 mg/mL, 12 h; 0.4 mg/mL, 12 h; control; 0.1 mg/mL, 24 h; 0.2 mg/mL, 24 h; 0.4 mg/mL, 24 h; control; 0.1 mg/mL, 48 h; 0.2 mg/mL, 48 h; 0.4 mg/mL, 48 h.

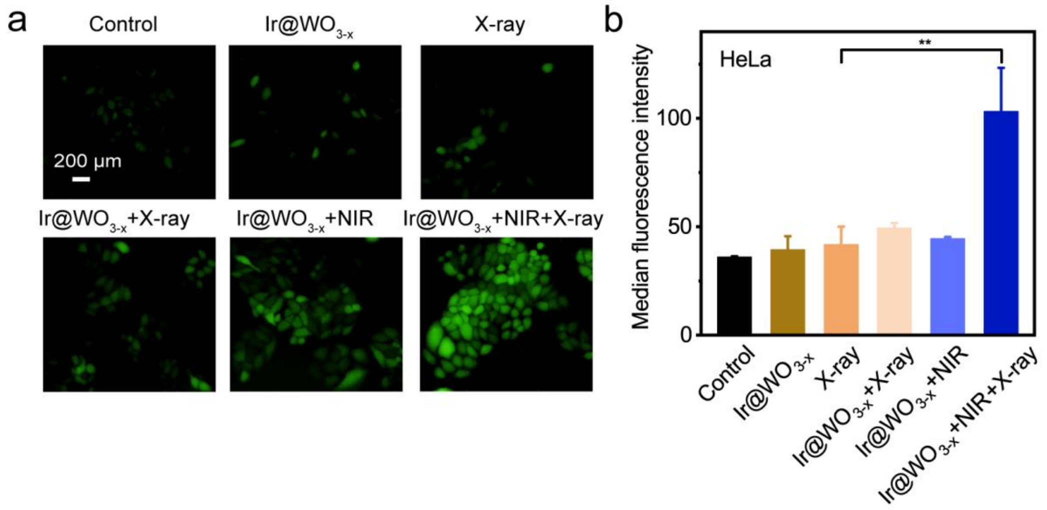

Figure S19. Fluorescence images (a) and statistical evaluation (b) of ROS produced by HeLa cells under various conditions (mean ± SD, n = 3). P values were assessed using a two-tailed Student's t test with GraphPad Prism 8.0. $^{**}P < 0.01$.

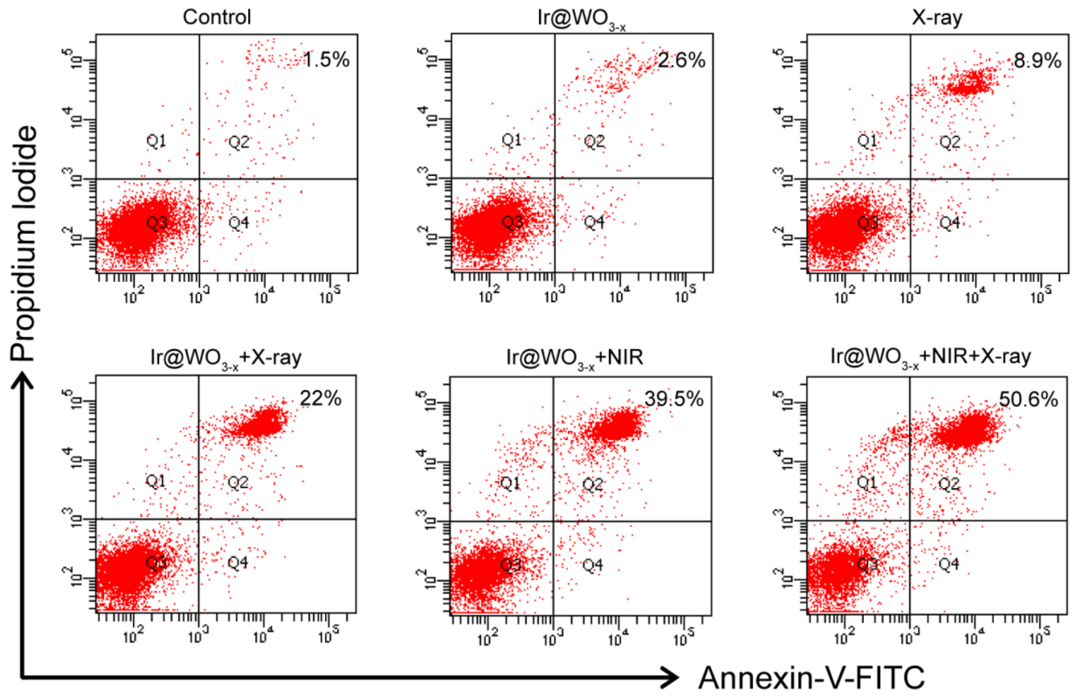

Figure S20. Annexin V-FITC/PI staining of HeLa cell apoptosis under various conditions.

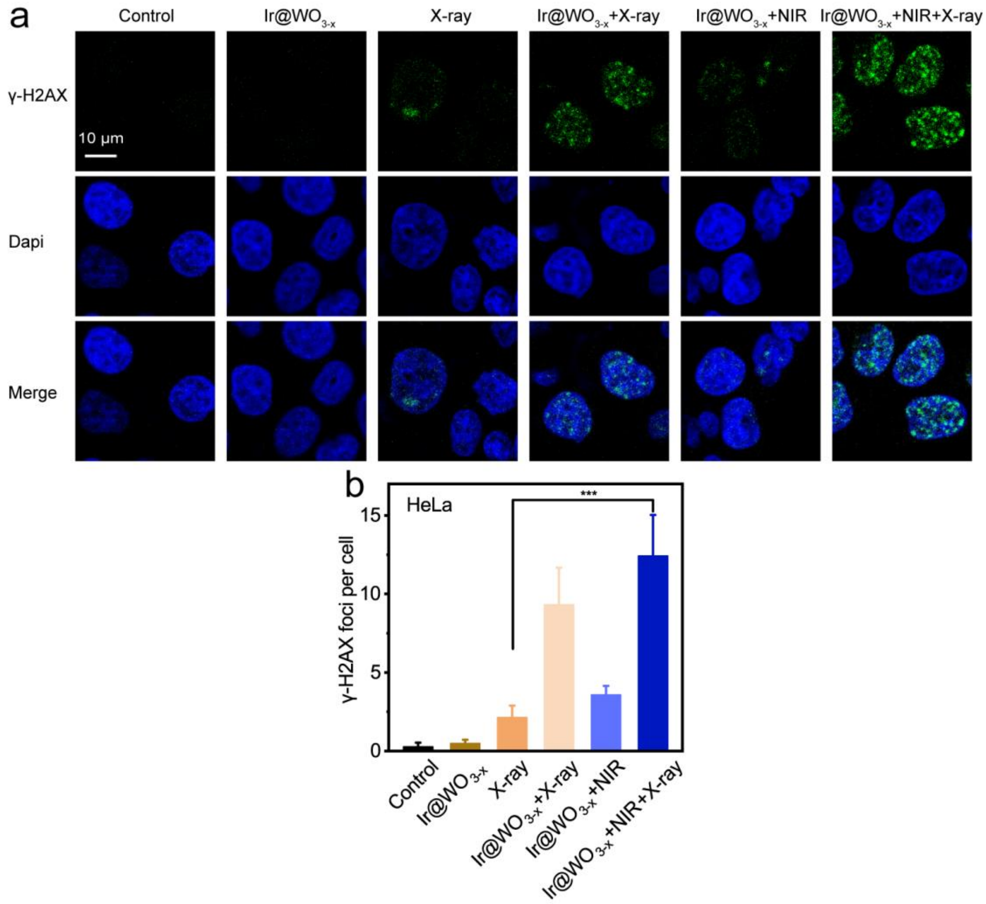

Figure S21. Fluorescence images (a) and statistical analysis (b) of γ-H2AX in HeLa cells in different treatment groups (mean ± SD, n = 3). P values were assessed using a two-tailed Student's t test with GraphPad Prism 8.0. ***$P < 0.001$.

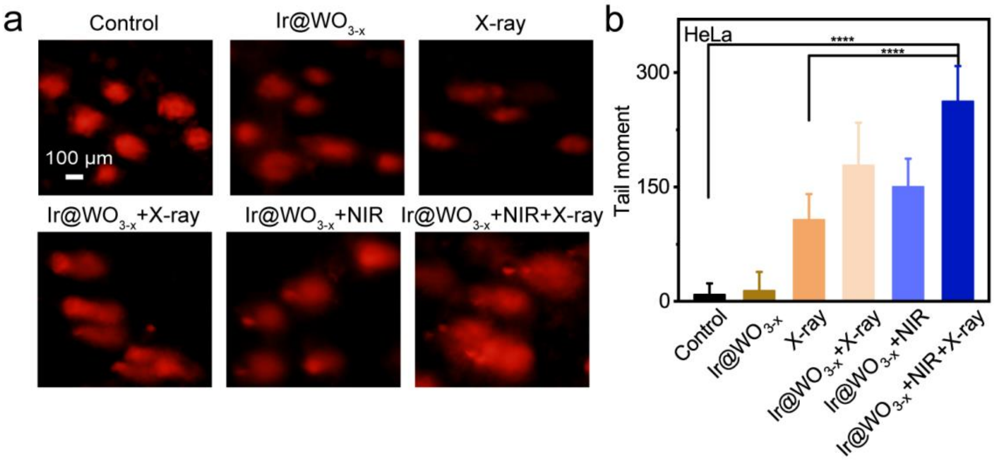

Figure S22. DNA damage in HeLa cells under various conditions, detected by comet assay (a), and quantitative analysis (b) (mean ± SD, n = 3). P values were assessed using a two-tailed Student's t test with GraphPad Prism 8.0. ****$P < 0.0001$.

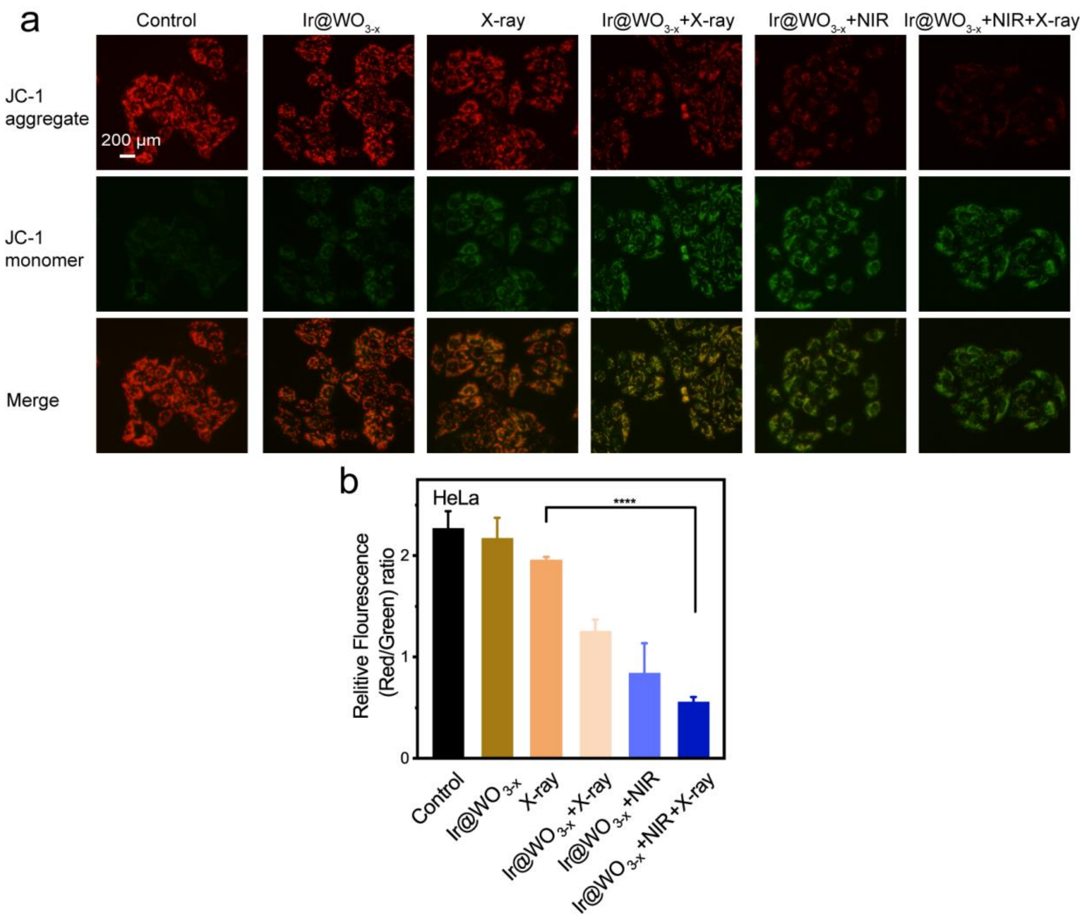

Figure S23. JC-1 staining of HeLa cell mitochondrial membrane potential (a) and quantitative analysis (b) for different treatment groups (mean ± SD, n = 3). P values were assessed using a two-tailed Student's t test with GraphPad Prism 8.0. ****$P < 0.0001$.

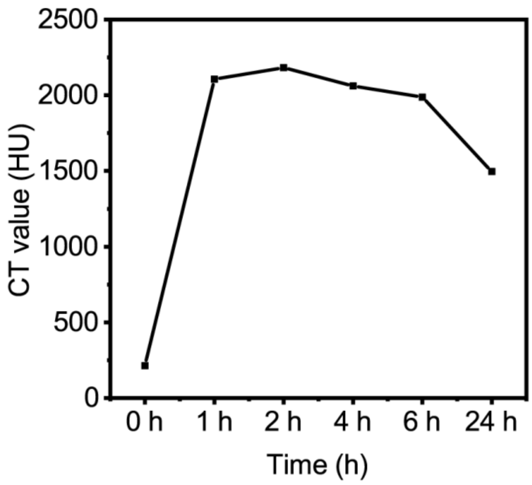

Figure S24. CT signal values at different time points.

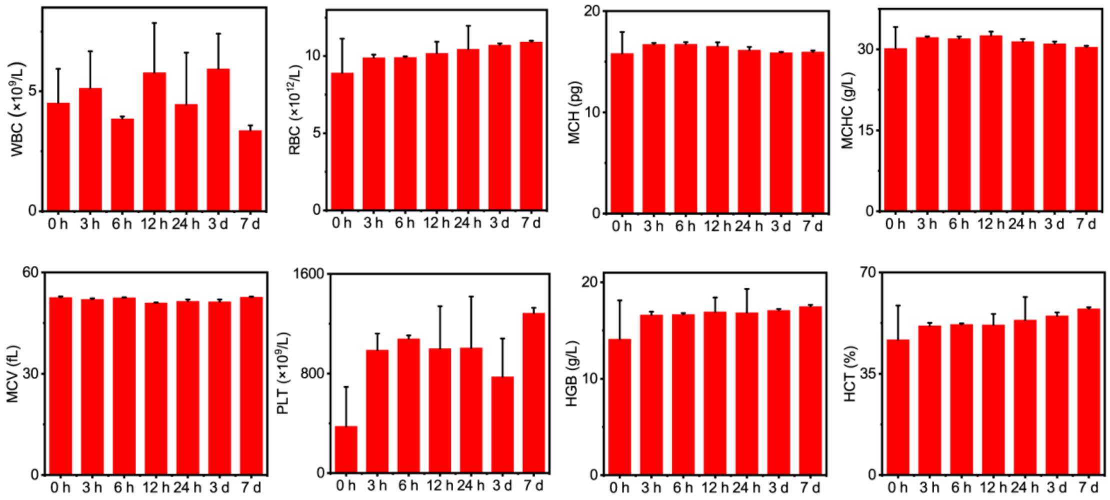

Figure S25. Blood parameters for mice injected with Ir@WO_{3-x} nanoreactors at different time points (mean ± SD, n = 3).

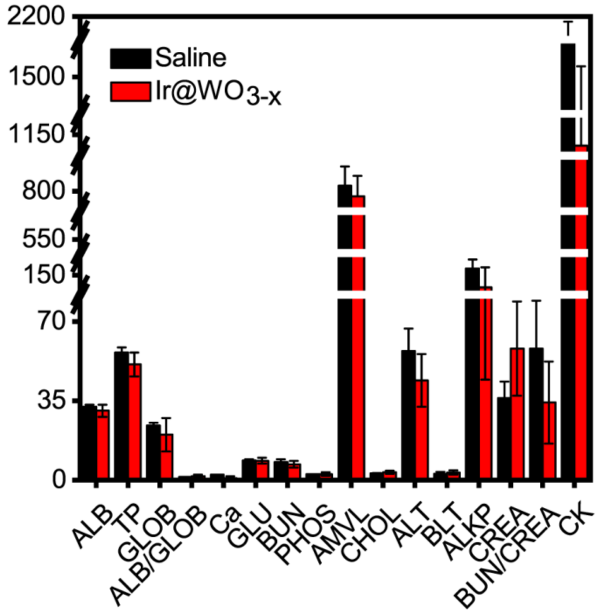

Figure S26. Biochemical analysis tests in mice 24 hours after Ir@WO_{3-x} injection (mean ± SD, n = 3).

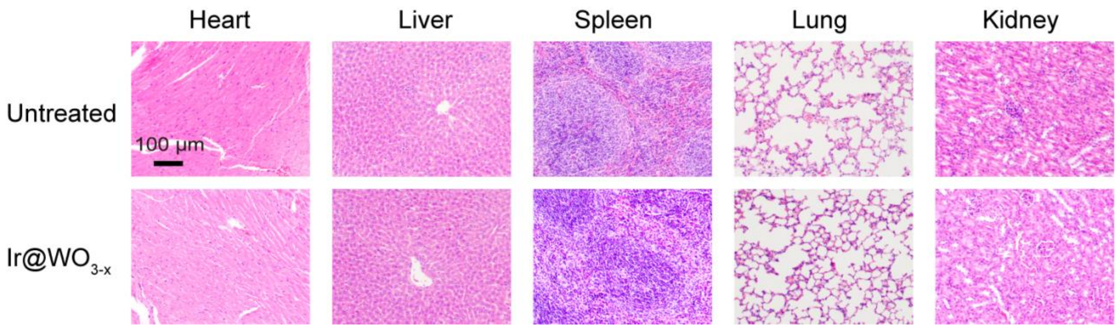

Figure S27. H&E staining of mouse organs on day 7 after Ir@WO_{3-x} nanoreactor injection.

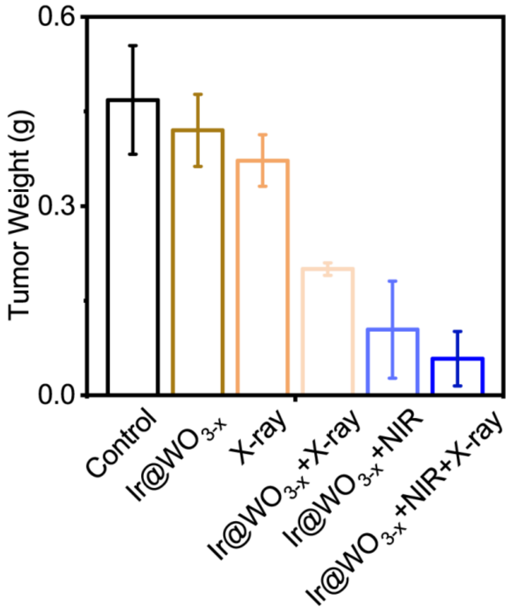

Figure S28. Tumor weight by treatment group (mean ± SD, n = 5).

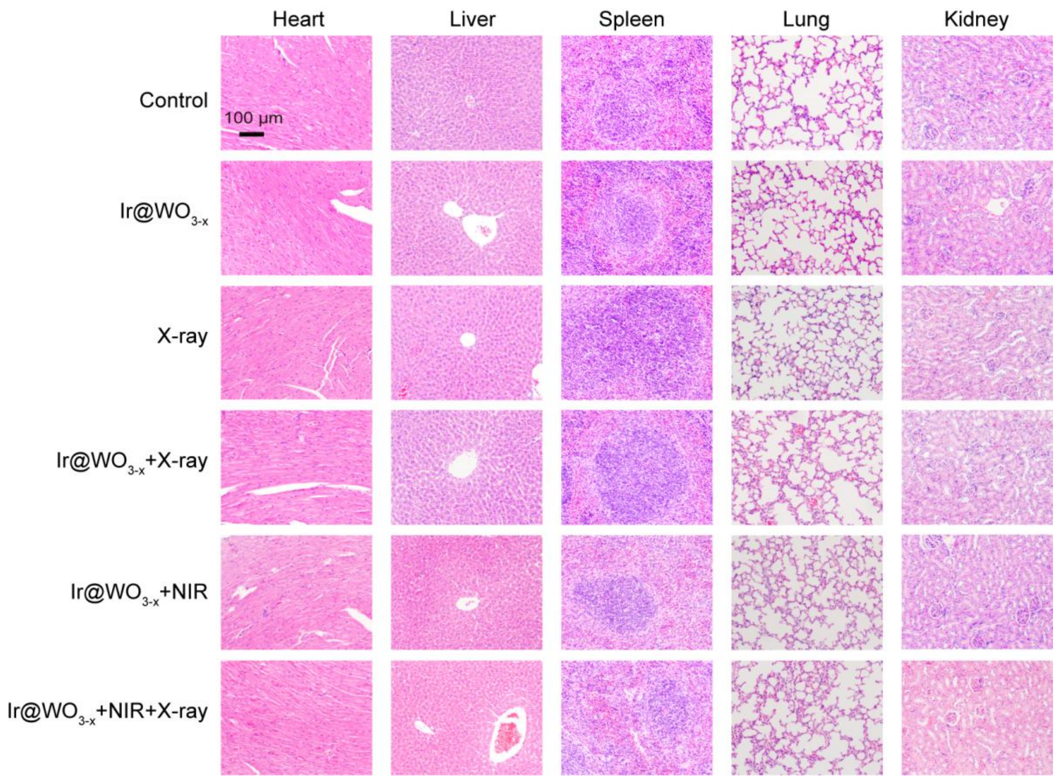

Figure S29. H&E-stained organ sections obtained 14 days after treatment.

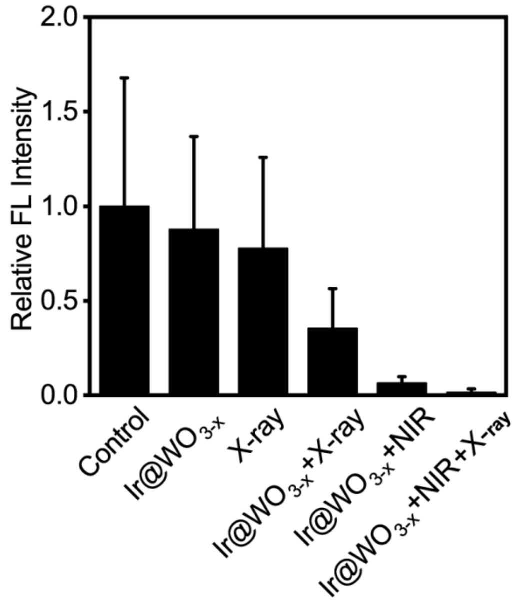

Figure S30. Tumor progression in the treatment groups at 28 days, observed in vivo by luciferase imaging (mean ± SD, n = 3).

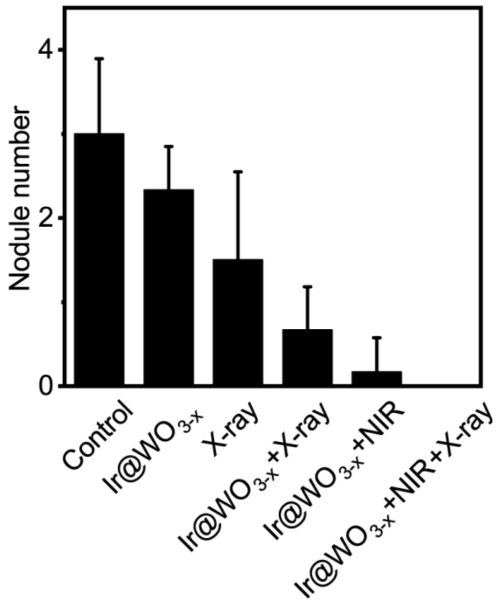

Figure S31. Lung tumor nodules after 28 days by treatment group (mean ± SD, n = 6).

Elment	O	W	Ir
Ir@WO_{3-x}	1	0.123	0.064

Table S1. Elemental composition of Ir@WO_{3-x} nanoreactors, determined by EDS.

Catalyst	[E] (mg/mL)	Substrate	K_M (mM)	V_{max} ($10^{-7}M*S^{-1}$)
Ir@WO_{3-x}	0.1	TMB	0.1304	66.869

Table S2. Kinetic parameters of the peroxidase-like activity of Ir@WO_{3-x} nanoreactors.
